# Genome‐Wide Analysis of *Tritipyrum HSFs* and Functional Characterization of *TtHSF97* for Salt Tolerance

**DOI:** 10.1002/fsn3.71418

**Published:** 2026-01-10

**Authors:** Xianjiao Qin, Wenzhen Li, Ruoruo Wang, Jianxia Xu, Yanqing Ding, Kuiyin Li, Mingjian Ren

**Affiliations:** ^1^ College of Agriculture Guizhou University Guiyang China; ^2^ Guizhou Institute of Upland Crops, Guizhou Academy of Agricultural Sciences Guiyang China; ^3^ Guizhou Key Laboratory of Biology and Breeding for Specialty Crops Guiyang China; ^4^ Guizhou Institute of Biotechnology, Guizhou Academy of Agricultural Sciences Guiyang China; ^5^ College of Agriculture Anshun University Anshun China

**Keywords:** heat shock transcription factors, molecular breeding, salt‐tolerance, sustainable agriculture, *tritipyrum*, *TtHSF97*

## Abstract

Soil salinization is a major abiotic stress constraining global agricultural production. In this study, a genome‐wide analysis of the salt‐tolerant hybrid germplasm *Tritipyrum* identified 103 heat shock transcription factors (*TtHSF*) genes, which were classified into three major groups (A, B, and C) and shared conserved motifs. Transcriptome data and qRT‐PCR revealed that 29 of the *TtHSF* genes exhibited high expression levels in response to salt stress and recovery. Notably, *TtHSF97*, localized on Tel5E01T611500, exhibited significantly upregulated expression under salt stress. Subcellular localization confirmed that TtHSF97 is a nuclear‐enriched protein, consistent with its function as a transcription factor. Heterologous overexpression of *TtHSF97* in *Arabidopsis* significantly enhanced salt tolerance of transgenic plants, enabling them to maintain a high leaf expansion rate and root length under 150 mM NaCl stress. Correlation analysis identified 733 genes positively correlated with *TtHSF97* expression, which are involved in metabolism, cellular processes, and stress responses. This study provides crucial genetic resources for improving the salt tolerance in major food crops through molecular breeding. The development of salt‐tolerant wheat varieties using *TtHSF97* will directly enhance crop productivity and ecological adaptability in saline soils, with significant application value for ensuring food security and promoting sustainable agricultural development. The results of this study demonstrate that transcription factor engineering is an effective strategy for improving crop stress resistance.

## Graphical abstract

This study focuses on the mechanism of heat shock transcription factor (*TtHSF*) mediated salt stress tolerance in *Tritipyrum*. A total of 103 *TtHSF* genes were identified through genome‐wide analysis, with the function of the *TtHSF97* being emphasized. It lays a solid foundation for the future breeding of crops with high salt tolerance via molecular breeding techniques, aiming to improve the productivity and ecological adaptability of crops grown in saline‐alkali soils, thereby contributing to the enhancement of global food security.

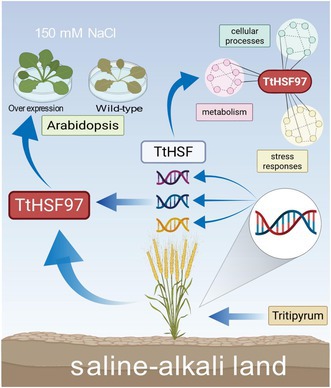

## 1 Introduction

Soil salinization represents one of the most severe abiotic challenges to global agriculture, affecting approximately 8.31 × 10^8^ hm^2^ worldwide, resulting in crop yield losses and incurring economic loss of up to $27.3 billion annually (Litalien and Zeeb 2020; Haj‐Amor et al. 2022; Muleta 2024). Saline soils disrupt plant growth via osmotic stress, ion toxicity, and oxidative damage induced by reactive oxygen species (ROS) that harm cellular components (Singh et al. 2022). In response, plants employ adaptive strategies spanning through morphological, physiological, and molecular scales to mitigate these effects (Yu et al. 2020; Tang et al. 2014; Wang et al. 2024; Segarra‐Medina et al. 2025). Notably, exploiting and utilizing salt‐tolerant germplasm remains the core approach to enhancing crop salt tolerance (Xu et al. 2017; Kumar et al. 2022; Yao et al. 2025). Central to orchestrating these complex salt tolerance responses is transcriptional reprogramming mediated by specific transcription factor families (Liang et al. 2018). Among these, Heat Shock Factors (HSFs) have emerged as pivotal regulators due to their indispensable roles in plant responses to environmental stresses.

HSFs facilitate plant adaptation to and defense against adverse environments by modulating the expression of a suite of stress‐responsive genes (Albihlal et al. 2018; Zang et al. 2019; Bakery et al. 2024). Studies have revealed that plant HSFs exhibit considerable variation in sequence size but share common structural features, which can be categorized into the following key conserved DNA binding domain and are phylogenetically categorized into three distinct classes (A, B, and C) (Döring et al. 2000; Treuter et al. 1993; Guo et al. 2016). Class A HSFs are primarily responsible for transcriptional activation, while class B and C HSFs are generally believed to lack this function (von Koskull‐Döring et al. 2007). HSFs exert their function by binding to heat shock elements (HSEs; 5′‐nGAAn‐3′) in target gene promoters, forming transcriptional complexes that drive heat shock protein (HSP) or other target genes' expression (Hu et al. 2015; Lin et al. 2011).

Initially identified in tomato under heat stress (Scharf et al. 1990), HSFs have been linked to a wide range of stress responses, including high and low temperatures, salinity, drought, light exposure, and disease. Most studies have focused on their role in thermal stress. In *Arabidopsis*, HSF members in class A are crucial in mediating the plant's response to heat stress (Bi et al. 2022; Ogawa et al. 2007), with specific subclass members, such as *HSFA1d* and *HsfA1e*, orchestrating the expression of downstream factors like *HsfA2*, thereby enhancing tolerance to combined high‐light and heat‐stress conditions (Nishizawa‐Yokoi et al. 2011). *AtHSFA6a*, *AtHSFA6b*, *AtHSFA9*, and *AtHSFC1* are markedly upregulated (> 40‐fold for *AtHSFC1*) under low‐temperature stress (Wang et al. 2024). Beyond temperature extremes, HSFs also exhibit broad functional diversity in stress adaptation. Expression profiling in *Arabidopsis* reveals that specific HSFs are significantly induced by distinct abiotic stresses. The expression of certain HSF genes in *Arabidopsis* was elevated, with *AtHSFA6a* expression showing a 146‐fold increase compared to the control under salt stress (Miller and Mittler 2006). *Arabidopsis* transgenic plants overexpressing the tomato *SlHsfA3* gene display enhanced thermotolerance but exhibit suppressed seed germination rates under salt stress, indicating a stress‐specific, potentially negative, regulatory role (Li et al. 2013). Conversely, *AtHSFA7b* functions as a positive regulator within the salt stress response pathway. It transcriptionally activates key components of the salt overly sensitive (SOS) signaling cascade–*AtSOS1*, *AtSOS2*, and *AtSOS3*–promoting cellular ion homeostasis and consequently enhancing salinity tolerance (Zang et al. 2019).

*T. aestivum* is moderately salt‐tolerant, outperforming rice ( *Oryza sativa* ) but lagging behind barley ( *Hordeum vulgare* ). *Th. elongatum* is closely related to *T. aestivum* and possesses the remarkable ability to thrive in salt concentrations akin to those found in seawater. The *octoploid Tritipyrum*, derived from intergeneric crosses between *T. aestivum* (AABBDD) and *Th. elongatum* (EE), serves as a significant source of germplasm for transferring salt tolerance genes from *Th. elongatum* into *T. aestivum* . The complete genome sequences available for both parents enable systematic dissection of stress‐responsive gene families, including *HSFs* (Mayer et al. 2014; Wang et al. 2020). However, despite the critical functions of *HSFs* in plant stress resistance and the significant value of *Tritipyrum* as a salt‐tolerant germplasm resource, there remains a clear gap in the comprehensive characterization of the *HSF* family in *Tritipyrum* (e.g., member composition, evolutionary relationships). This gap not only hinders in‐depth understanding of the genetic basis of salt tolerance in *Tritipyrum* but also becomes a key bottleneck restricting the efficient transfer of its salt tolerance genes to common wheat, thereby impeding efforts to enhance global agricultural production on saline‐alkali lands.

This study aimed to determine whether specific *HSF* genes in *Tritipyrum* contribute to enhanced salt tolerance by regulating ion homeostasis and stress signaling through regulatory networks. To this end, the genomic structural features, chromosomal locations, gene duplications, and evolutionary divergence of the *HSF* gene family in *Tritipyrum* were examined. Subsequently, the expression profiles of 29 *TtHSF* genes induced by salt stress were analyzed. Pearson correlation analyses and expression levels of *TtHSF97* during both salt stress and recovery phases were determined. Then subcellular localization and heterologous overexpression assays in *Arabidopsis* assessed its role in salt tolerance. These insights will advance our understanding of HSF‐mediated salt tolerance mechanisms and inform molecular breeding strategies for enhancing crop resilience in saline environments.

## 2 Materials and Methods

### 2.1 Plant Material

*Tritipyrum*, a cross bred octoploid, integrates the A, B, and D genomes of *T. aestivum* with the E genome of *Th. elongatum*. The salt‐tolerant *Tritipyrum* line ‘Y1805’, a stable progeny from a wide cross between *T. aestivum* and *Th. elongatum*, was used in this study (Tian et al. 2024). Protein and nucleic acid sequences from *Tritipyrum*, utilized for identifying *HSF* genes, were retrieved from the genome databases of *T. aestivum* and *Th. elongatum* (http://plants.ensembl.org, Project number: PRJNA361927 and PRJCA002191). Comparative genomic data from *Arabidopsis*, *Hordeum vulgare* , *Oryza sativa* , *Zea mays* , and *Thinopyrum intermedium* via Phytozome 13 (https://phytozome‐next.jgi.doe.gov/), while *S. cereale* genome sequences were retrieved from the China National GeneBank Database (https://ngdc.cncb.ac.cn). Publicly available transcriptome data were analyzed, accessible at NCBI BioProject (https://www.ncbi.nlm.nih.gov/bioproject/PRJNA769794), with an Accession number of PRJNA769794.

### 2.2 Identification of the *HSF* Genes in *Tritipyrum*

The consensus protein sequences for the HSF hidden Markov model (HMM) (PF00447) were retrieved from the Pfam database (http://www.pfam.sanger.ac.uk/). TtHSF protein candidates were identified using HMMER3.0 with default settings, and a threshold value of 0.01 was applied for elimination. Additionally, a search library was created from 21 previously reported AtHSF sequences, which were sourced from the UniProt database (www.uniprot.org). By employing the published sequences of HSF proteins from *Arabidopsis* and their corresponding HSF domain as query sequences, we conducted a search for TtHSF proteins using the BLASTP algorithm. After removing duplicates, candidate sequences were validated using the Pfam database and the SMART tool (http://smart.embl‐heidelberg.de/; Letunic et al. 2012). The physicochemical properties of the identified *TtHSF* genes were analyzed using ExPASy (http://web.expasy.org/protparam/).

### 2.3 Phylogenetic Analyses and Conserved Motif Determination

To investigate the evolutionary relationships of *TtHSF* genes, *Arabidopsis* HSF protein sequences were retrieved from UniProt (https://www.uniprot.org). Amino acid sequences of identified *TtHSF* genes were aligned using ClustalX v2.0. Phylogenetic trees were constructed for *Tritipyrum* and *Arabidopsis* using the Neighbor‐Joining method with the Poisson model and 1000 bootstrap replicates in MEGA software. Conserved motifs in TtHSF proteins were identified using the MEME Suite (https://meme‐suite.org/meme/) with motif widths set between 6 and 200 and a maximum of 10 motifs. Phylogenetic trees were visualized and annotated using FigTree v1.4.4 and iTOL (https://itol.embl.de/).

### 2.4 Chromosomal Distribution and Gene Duplication of the *TtHSF* Genes

The chromosomal locations of *TtHSF* genes to the chromosomes of *Tritipyrum* were mapped using the approach described by Liu et al. (2014). Gene duplication events were analyzed with the MCScanX toolkit v1.0 using default parameters (Buchfink et al. 2015). All‐versus‐all protein sequence comparisons required for MCScanX were performed using DIAMOND v0.8.25 with parameters ‐max‐target‐seqs 5 and ‐*e* value 0.00001.

### 2.5 *Tritipyrum* Growth Conditions and Stress Treatments

Seeds of *Tritipyrum* ‘Y1805’ were germinated in a controlled chamber at 75% relative humidity with a 20°C/15°C light/dark cycle. Seedlings were cultivated in 1/2 Hoagland's solution on a floating board under a 16/8‐h light/dark cycle, 400 μmol m^−2^ s^−1^ irradiance, and consistent temperature and humidity conditions. The nutrient solution was refreshed three times weekly. On day 14 (two‐leaf stage), salt stress was induced using 1/2 Hoagland's solution with 250 mM NaCl. Root, stem, and leaf samples of uniform size from *Tritipyrum* (T_1_) were collected 5 h after exposure to salt stress. After 24 h of stress, plants were transferred to 1/2 Hoagland's solution without NaCl, and a second sample (T_2_) was collected 1 h after recovery. Control groups (CK_1_ and CK_2_) were maintained in 1/2 Hoagland's solution without NaCl. Expression level samples were snap‐frozen in liquid nitrogen and stored at −80°C. Each sample pooled at least ten seedlings from three biological replicates.

### 2.6 Expression Analysis and qRT‐PCR Validation of *HSF* Genes Under Salt Stresses and Recovery

RNA‐Seq datasets were preprocessed using fastp v0.2 to remove adaptor contamination, low‐quality bases, and undetermined bases with default parameters. High‐quality reads were aligned to the combined genomes of *T. aestivum* v2.0 and *Th. elongatum* v1.0 using Bowtie v2.2.3 and HISAT v2.2.0. Read counts for *Tritipyrum HSF* genes were quantified using FeatureCounts v1.5.1 (Liao et al. 2014). Expression levels, calculated as transcripts per kilobase million (TPM), were log‐transformed and visualized using the R Circlize package (https://cran.r‐project.org/web/packages/circlize.html). Functional gene annotation was performed through GO annotation by mapping GO and KEGG terms using the BLAST2GO tool (http://blast2go.com/b2ghome/about) with an e‐value threshold of 10^−6^. qRT‐PCR primers were designed using Primer v5.0 (Table S2). The *Actin* gene served as the internal control, consistently expressed across growth stages and tissues. Expression levels were determined using the 2^−ΔΔCt^ method with three technical replicates from three biological replicates.

### 2.7 Vector Construction and Agrobacterium‐Mediated Transformation in *Arabidopsis*

The coding sequence (CDS) of *TtHSF97* was amplified from Y1805 seedling cDNA using gene‐specific primers TtHSF97‐CDS‐XbaI (GCTCTAGAGCATGGACCCCTTCCACGGC) and TtHSF97‐CDS‐KpnI (GGGGTACCCCTCACTGGTGGCTGTGGGGC). The resulting PCR product was digested with *XbaI* and *KpnI* restriction enzymes and ligated into the similarly digested *pCMBIA3301* vector to construct the overexpression vector *pCMBIA3301‐TtHSF97*. This recombinant plasmid was introduced into *Arabidopsis* ecotype Col‐0 via *Agrobacterium tumefaciens* strain EHA105. Transgenic T_1_ seeds were surface‐sown on half‐strength MS medium supplemented with 15 mg·L^−1^ Basta for antibiotic selection. Resistant seedlings exhibiting true leaf development and root formation were transplanted and propagated to the T_3_ generation to obtain homozygous transgenic lines. *TtHSF97* expression levels were quantified by qRT‐PCR. Two independent transgenic lines with the highest expression levels were selected for subsequent functional studies.

### 2.8 Salt Stress Tolerance Phenotyping During Early Development in *Arabidopsis*

Seeds of Col‐0 and two *TtHSF97*‐overexpressing lines (#1 and #6) were divided into two groups: a control group grown under standard conditions and a salt‐stress group sown on 1/2 MS solid medium supplemented with 150 mM NaCl. All seeds were in a controlled‐environment growth chamber (25°C, 60% RH, 16/8 h light/dark cycle). On day 14, the green true leaves expansion rate (%) was quantified using the formula: (Number of seedlings with fully expanded true leaves/Total seedlings per line) × 100%. At the same time, seeds were plated on 1/2 MS medium (normal) for 1 week and then plated on 1/2 MS or 1/2 MS medium containing 150 mM NaCl for 1 week for comparing the root length. Three biological replicates were performed per genotype‐treatment combination, with each replicate containing ≥ 50 seeds randomized across plates.

### 2.9 Subcellular Localization of TtHSF Protein

The open reading frame (ORF) of *TtHSF97* was amplified from a Y1805 seedling cDNA library using gene‐specific primers TtHSF97‐GFP‐F (CTCAAGCTTGGATCCATGGACCCCTTCCACGGC), and TtHSF97‐GFP‐R (GCTCACCATACTAGTCTGGTGGCTGTGGGGC). The resulting PCR product was digested and inserted into the same sites in the *pHB* plasmid (Mao et al. 2005) to generate the recombinant construct *2 × 35S::TtHSF97‐CDS‐GFP*. This plasmid was subsequently transformed into *A. tumefaciens* strain GV3101 using the freeze–thaw method (Weigel and Glazebrook 2006). Bacterial cultures were prepared for infiltration by resuspending cells in an induction buffer. For transient expression, 1 mL of the bacterial suspension was pressure‐infiltrated into the abaxial epidermis of *Nicotiana benthamiana* leaves using a needleless syringe, with infiltrated zones clearly marked. After 48 h of low‐light incubation, GFP fluorescence was visualized and imaged using a laser scanning confocal microscope (Leica TCS SP5, Germany). For confocal imaging, GFP was excited at 488 nm and emission was collected at 507 nm, while mCherry was excited at 543 nm with emission collected at 568 nm. The subcellular localization assay was performed in three independent biological replicates. For each replicate, at least 100 cells were observed per field, and consistent localization patterns were obtained across all repeats, confirming good experimental reproducibility. Control assays were performed in parallel using *A. tumefaciens* transformed with the empty *pHB* vector.

### 2.10 Statistical Analysis

The study data were analyzed using analysis of variance (ANOVA) with SPSS software (IBM Corporation, version 28.0). The least significant difference (LSD) test was applied to compare mean values at a significance threshold of 0.05. Histograms were generated using Origin 8.0 (OriginLab Corporation, Northampton, Massachusetts, USA).

## 3 Results

### 3.1 Identification of the *TtHSF* Genes in *Tritipyrum*

**FIGURE 1:**
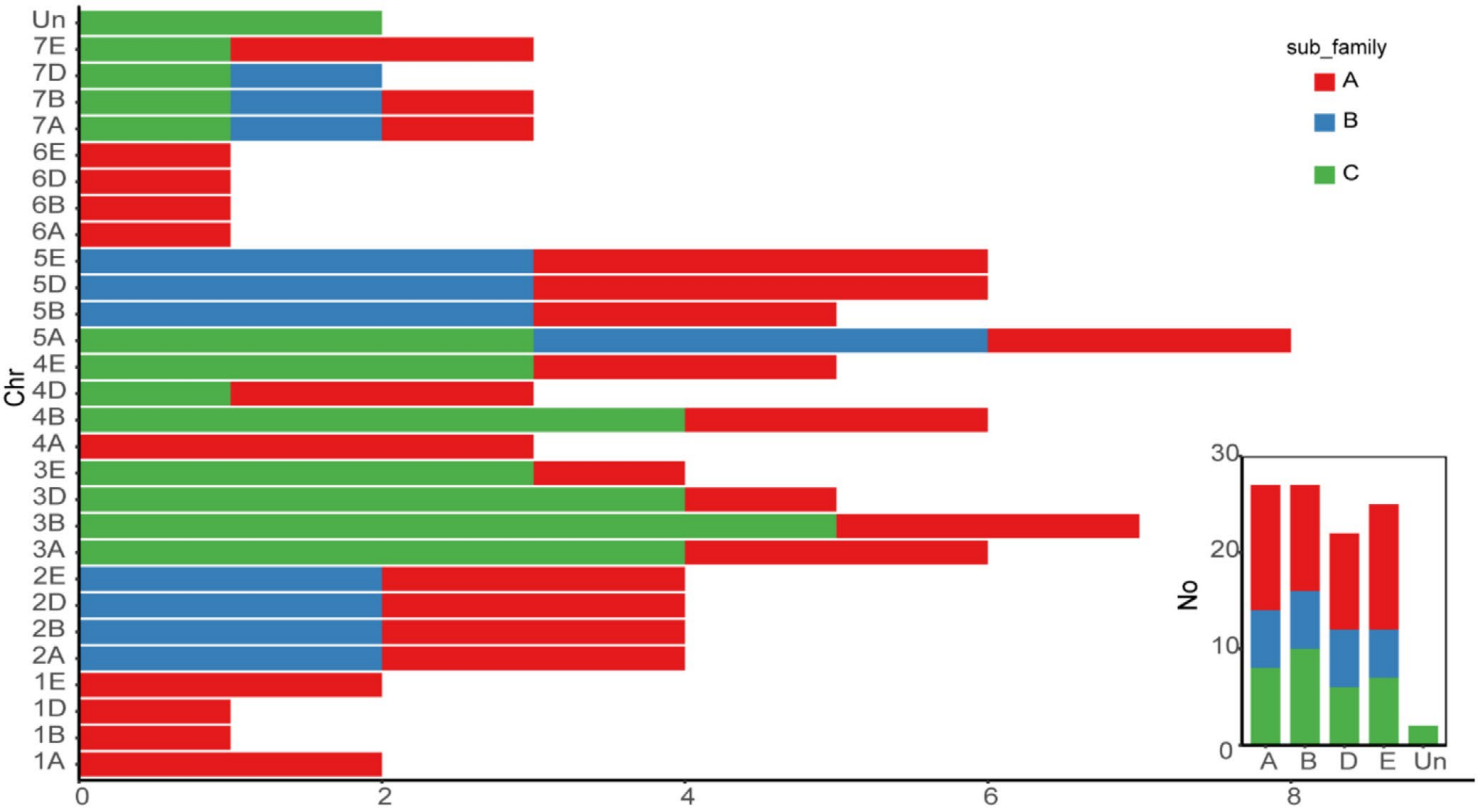
Chromosomal and subgenomes distribution of *TtHSF* genes in *Tritipyrum*.

After removing duplicated sequences, 103 *HSF* genes were identified in the *Tritipyrum* genome using both the HMM and BLASTp approaches in this study, and were renumbered according to their positions on *Tritipyrum* chromosomes (Table S1). The analysis revealed that the 103 identified *HSF* genes in *Tritipyrum* were predominantly distributed in the A and B subgenomes (Figure 1), encoding proteins ranging from 145 to 569 amino acids in length (e.g., Tel4E01T582800.1 to TraesCS5B02G315600.1), and from 16.6 to 59.75 kDa in molecular weight (e.g., Tel4E01T582800.1 to TraesCS5B02G315600.1). The predicted *TtHSF* proteins exhibited considerable variation in both length and molecular weight (MW). The isoelectric points (PIs) of the proteins varied from 4.51 (Tel7E01T619800.1) to 9.97 (TraesCS5B02G236400.1; Table S1).

### 3.2 Phylogenetic Analysis, and Motif Composition of the TtHSFs

**FIGURE 2:**
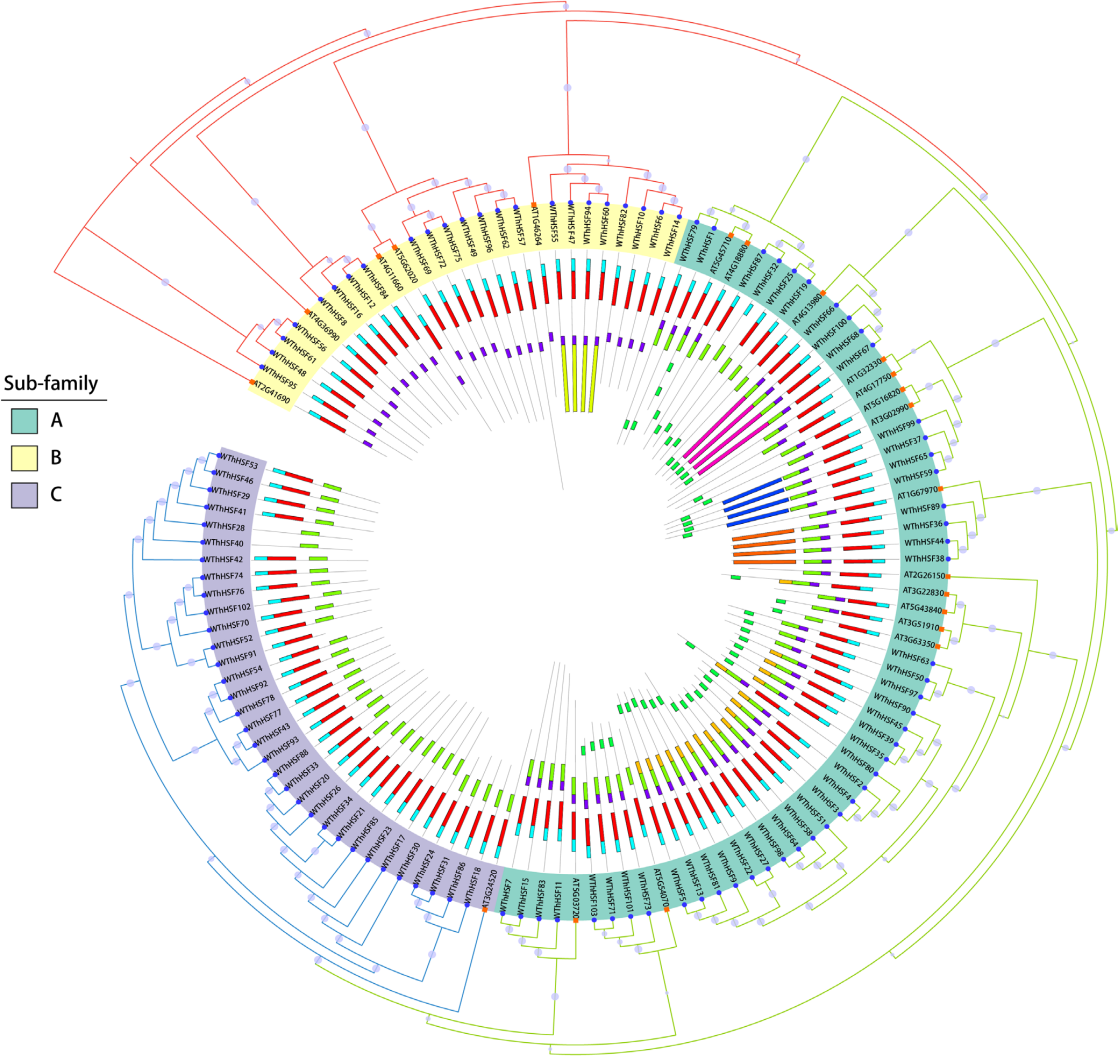
Phylogenetic relationships of the HSF proteins from *Tritipyrum* and *Arabidopsis*.

To explore the evolutionary relationships and classify the HSF family in *Tritipyrum*, 124 potential HSF proteins identified in *Arabidopsis* and *Tritipyrum* were employed to construct a phylogenetic tree (Figure 2). Based on the primary amino acid sequence features and the classification of AtHSFs, the *Tt*HSF family members were classified into three major groups. Group A, the largest subfamily, contained 47 proteins, whereas the smallest, Group B, comprised only 23 proteins. *M*ost *TtHSF* proteins within the same group shared conserved motifs (Figures 2 and S1). Furthermore, the distribution of conserved amino acids within the HSF domains of *Tritipyrum* closely resembled that of *Arabidopsis*, suggesting that HSFs have been conserved throughout plant evolution. The factors of the C subfamily were highly conserved between *Tritipyrum* and *Arabidopsis*. Subgroup B displayed greater variation than subgroup C. *Tritipyrum* and *Arabidopsis* displayed a smaller genetic divergence between A and C (Figure 2). Genetic distance analysis within and between HSF subfamilies further supported this phylogenetic classification (Figure S2).

### 3.3 Chromosomal Distribution, Gene Duplication and Synteny Analysis of the *TtHSFs*

**FIGURE 3:**
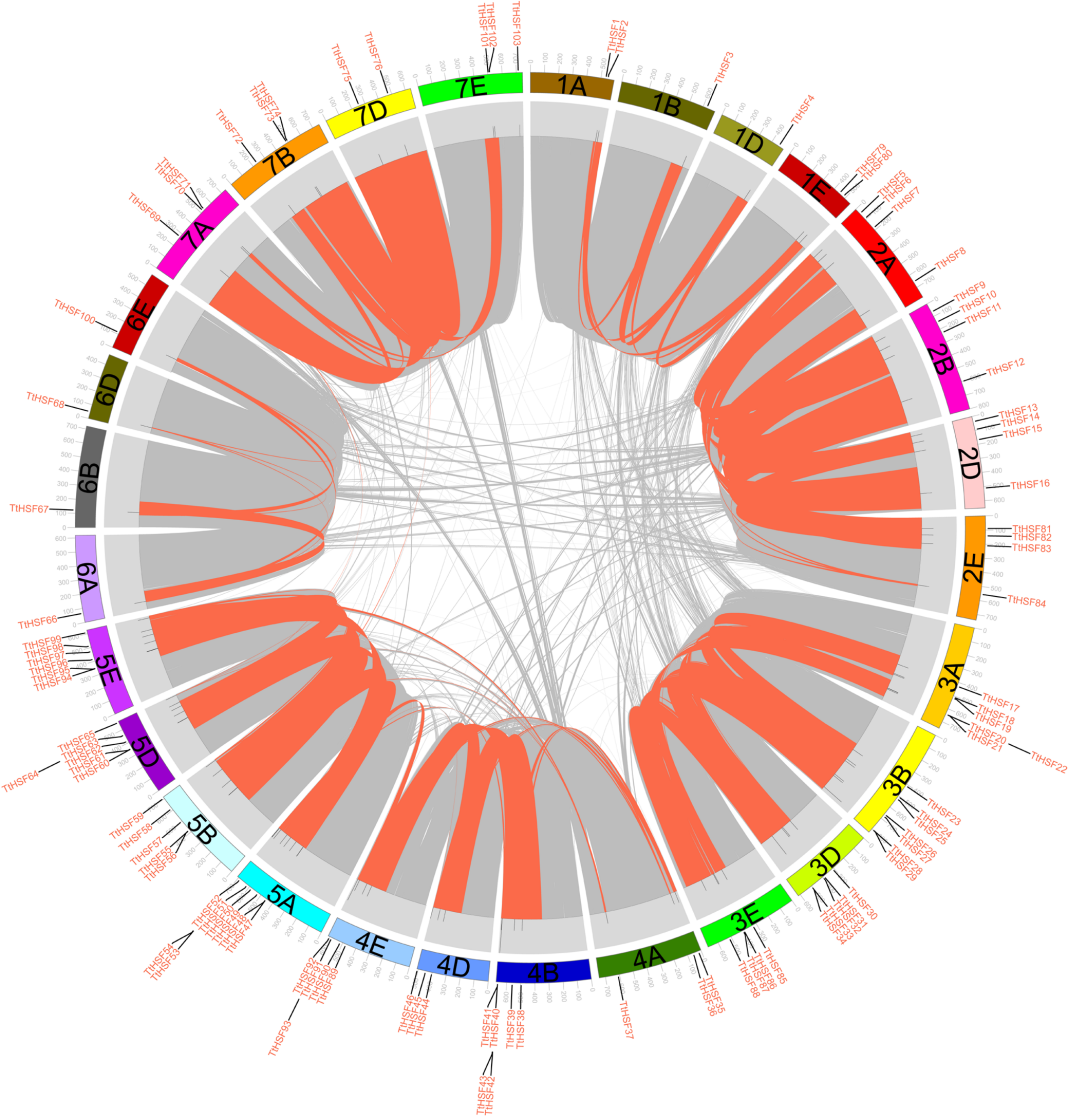
Distribution, duplication, and synteny analysis of *TtHSF* genes in *Tritipyrum*. The collinear correlations of *TtHSF* genes in *Tritipyrum* genomes are visualized using a Circos plot. *Tritipyrum* chromosomes are color‐coded according to their inferred ancestral origins, following established conventions. In the central part of the plot, the positions of annotation genes across all 28 *Tritipyrum* chromosomes are displayed, with 101 *TtHSF* genes highlighted in color.

Out of the 103 *TtHSF* genes, 101 were mapped to 28 chromosomes, while only *TtHSF77* and *TtHSF78* were excluded from the chromosome localization map due to their placement on scaffolds (Table S1, Figure 3). Most *TtHSF* genes were distributed across the third (21.3%) and fifth (24.3%) homologous clusters, whereas fewer were found in the first (5.8%) and sixth (3.9%) homologous groups (Table S1, Figures 1 and 3). Based on their chromosomal positions, most *TtHSF* genes were situated towards the chromosomal ends, with only a few near the center (Figure 3). The previous results indicated that the distribution of plant *HSF* genes across chromosomes was non‐random and uneven. Furthermore, an analysis of gene duplication showed that *TtHSF* genes experienced tandem duplications, with 12 pairs of tandemly duplicated genes identified (Figure 3). This indicates that certain genes have multiple copies, likely resulting from several rounds of replication within the *T. aestivum* genome. The majority of homologous genes are distributed within the same homologous groups, with only a few located in the fourth, fifth, and seventh groups, aligning with the natural translocations during the development and evolution of *T. aestivum* .

### 3.4 Evolutionary Analysis of the *HSF* Families in Several Different Species

**FIGURE 4:**
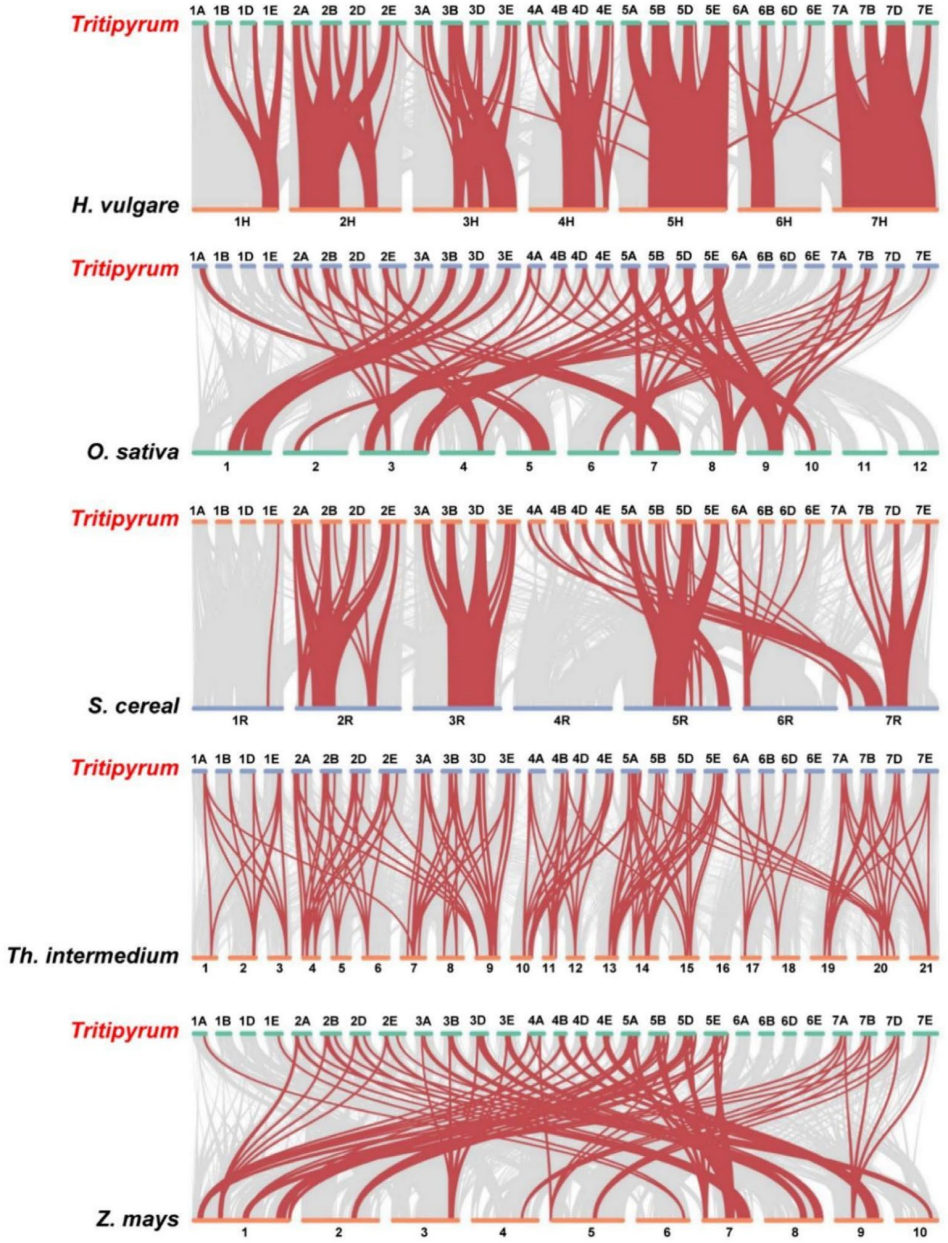
Synteny analyses of the *HSF* genes between *Tritipyrum* and five representative plant species. Background lines indicate inter‐genomic collinear blocks within *Tritipyrum* and other plant genomes, while red lines highlight syntenic *TtHSF* gene pairs.

To further deduce the evolutionary ties between *HSF* gene family members in *Tritipyrum*, *H. vulgare* , *O. sativa* , *S. cereale* , *Th. intermedium*, and *Z. mays* , the syntenic relationships between the six species were investigated (Figure 4). 102 *TtHSF* genes shared syntenic connections with those of *Th. intermedium*, followed by *Z. mays* (53), *S. cereale* (50), *O. sativa* (45), and *H. vulgare* (44) (Figure 4). Collinear pairs were identified between *Tritipyrum* and the other five species, indicating that these orthologous pairs likely predated the ancestral divergence event. Additionally, *HSF* collinear gene pairs found between *Tritipyrum* and *H. vulgare* were anchored to the highly conserved syntenic blocks, which encompassed over 500 collinear loci. Comparable syntenic conservation was observed between *Tritipyrum* and *S. cereale* , potentially reflecting their shared evolutionary trajectory among the six plant species. Significantly, multiple *TtHSF* genes were associated with at least three syntenic gene pairs, implying a potentially key role for these genes in the evolutionary diversification of the *HSF* gene family. These findings support high conservation within the *TtHSF* gene family and indicate greater phylogenetic proximity to *Z. mays HSFs* than to those of *H. vulgare* . Collectively, these data suggest that the *TtHSF* genes likely originated from a monophyletic ancestor within the monocotyledon lineage.

### 3.5 Expression of *TtHSF* Genes Under Salt Stresses and Recovery

**FIGURE 5:**
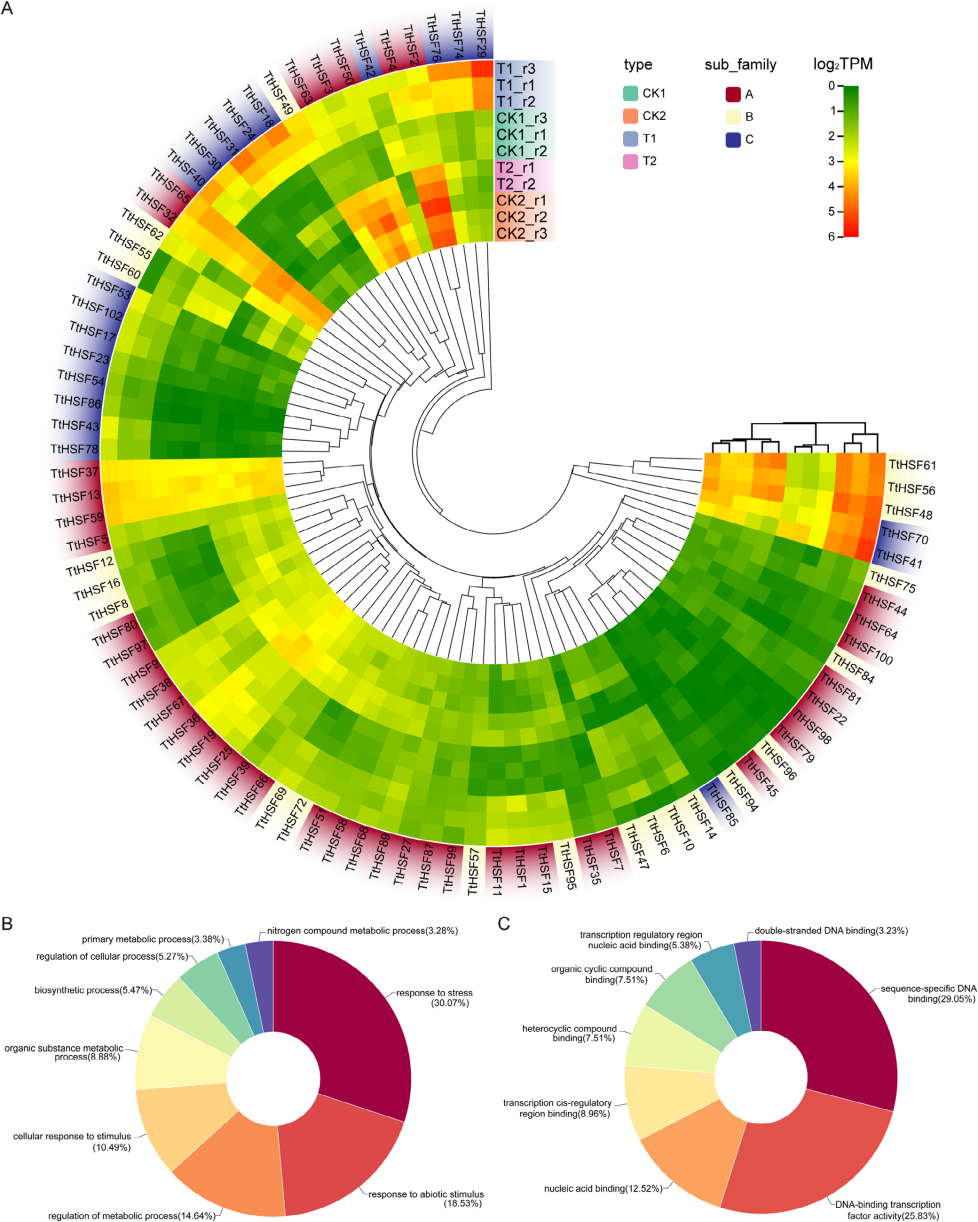
Expression patterns and functional annotation of *TtHSF* genes under salt stress and recovery treatment. (A) Hierarchical clustering of 84 expressed *TtHSF* genes across 11 samples including salt stress and recovery treatment. Colored branches indicate expression subclusters with no significant correlation to gene subfamilies. (B and C) The Enriched BP (B) and MF (C) from GO analysis.

**FIGURE 6:**
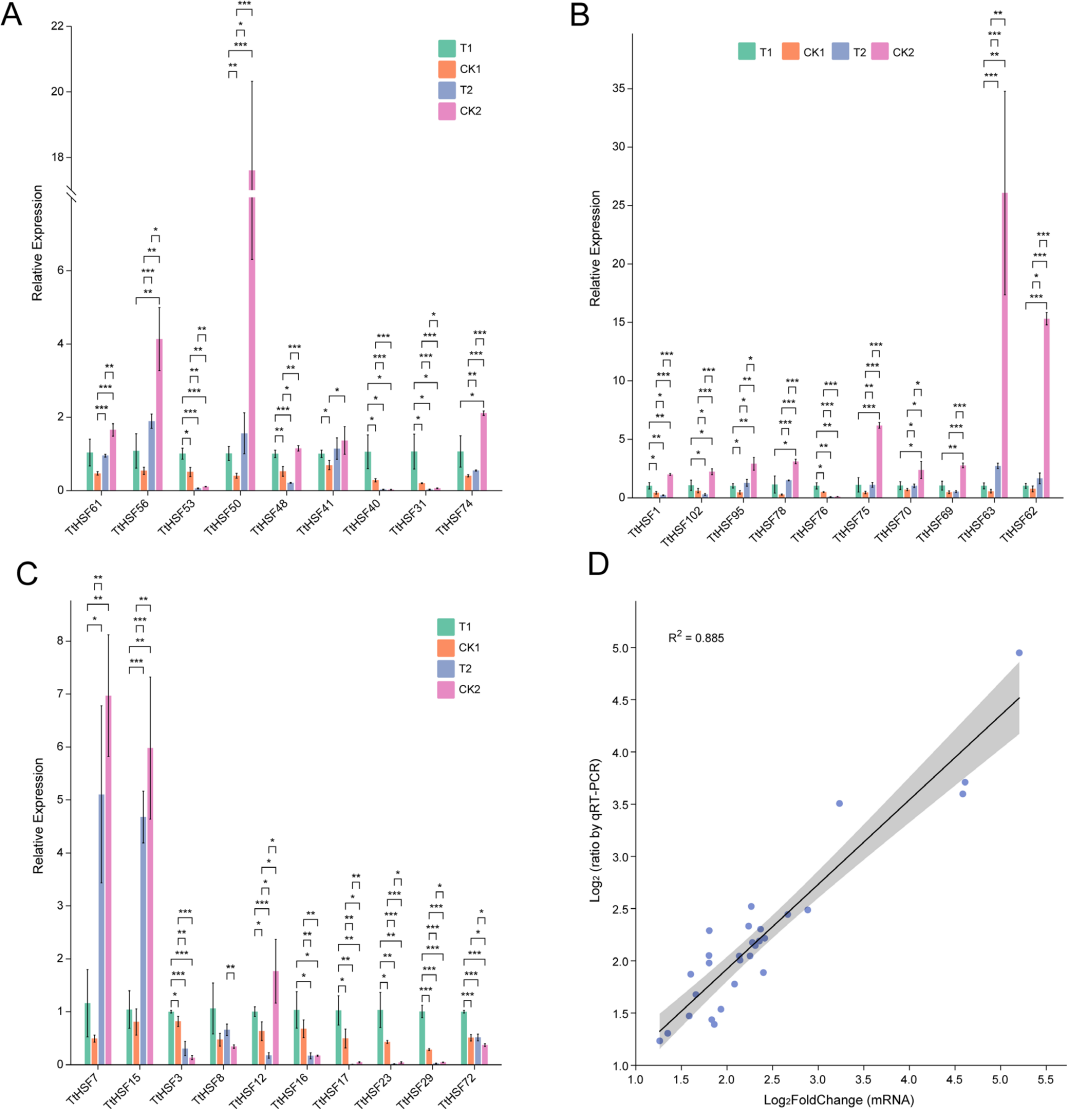
Expression analysis and correlation of *TtHSF* genes in response to salt stress. (A–C) Expression analysis of 29 *TtHSF* genes across 11 samples using qRT‐PCR. Gene expression levels were normalized to the *β‐actin* gene, and vertical bars represent the standard deviation. (D) Correlation analysis between qRT‐PCR and transcriptional data for the 29 up‐regulated genes. Expression values are presented as the log₂ ratio (salt stress or recovery treatment vs. CK treatment). The coefficient of determination (*r* ^2^) is shown in the figure. All qRT‐PCR experiments were performed in three biological replicates. *p < 0.05; **p < 0.01; ***p < 0.001.

To evaluate transcriptional dynamics of *TtHSF* gene under varying salt regimens, we profiled transcript abundances of all 103 *TtHSF* genes across salt stress conditions and post‐stress recovery phases. Among these, 84 *TtHSF* genes were found to be expressed in all 11 samples (TPM > 0), with 41 exhibiting constitutive expression. Hierarchical clustering revealed no significant association between gene subfamily classification and expression responsiveness to salt stress or recovery treatments (Figure 5A). Nineteen *TtHSF* genes were not detected in any of the samples, implying that they could be pseudogenes or stress‐nonresponsive loci. Gene Ontology (GO) annotation of the 84 expressed genes enriched biological processes (BP) including response to stress, response to abiotic stimulus, regulation of metabolic process, and cellular response to stimulus, along with molecular functions (MF) such as sequence‐specific DNA binding, transcription factor activity, nucleic acid binding, cis‐regulatory region binding, and binding of heterocyclic compounds (Figure 5B,C). To further confirm the accuracy of the transcriptome data, we selected 29 *TtHSF* genes with differential expression (|log_2_FoldChange| > 1) across treatments for qRT‐PCR confirmation using gene‐specific primers (Table S2). The results from qRT‐PCR and transcriptome data of the selected genes showed a high degree of correlation (*r* ^*2*^ = 0.885; Figure 6A–C), confirming the validity of our transcriptomic analysis (Figure 6D).

### 3.6 Functional Characterization of *TtHSF97* in Salt Stress Response

**FIGURE 7:**
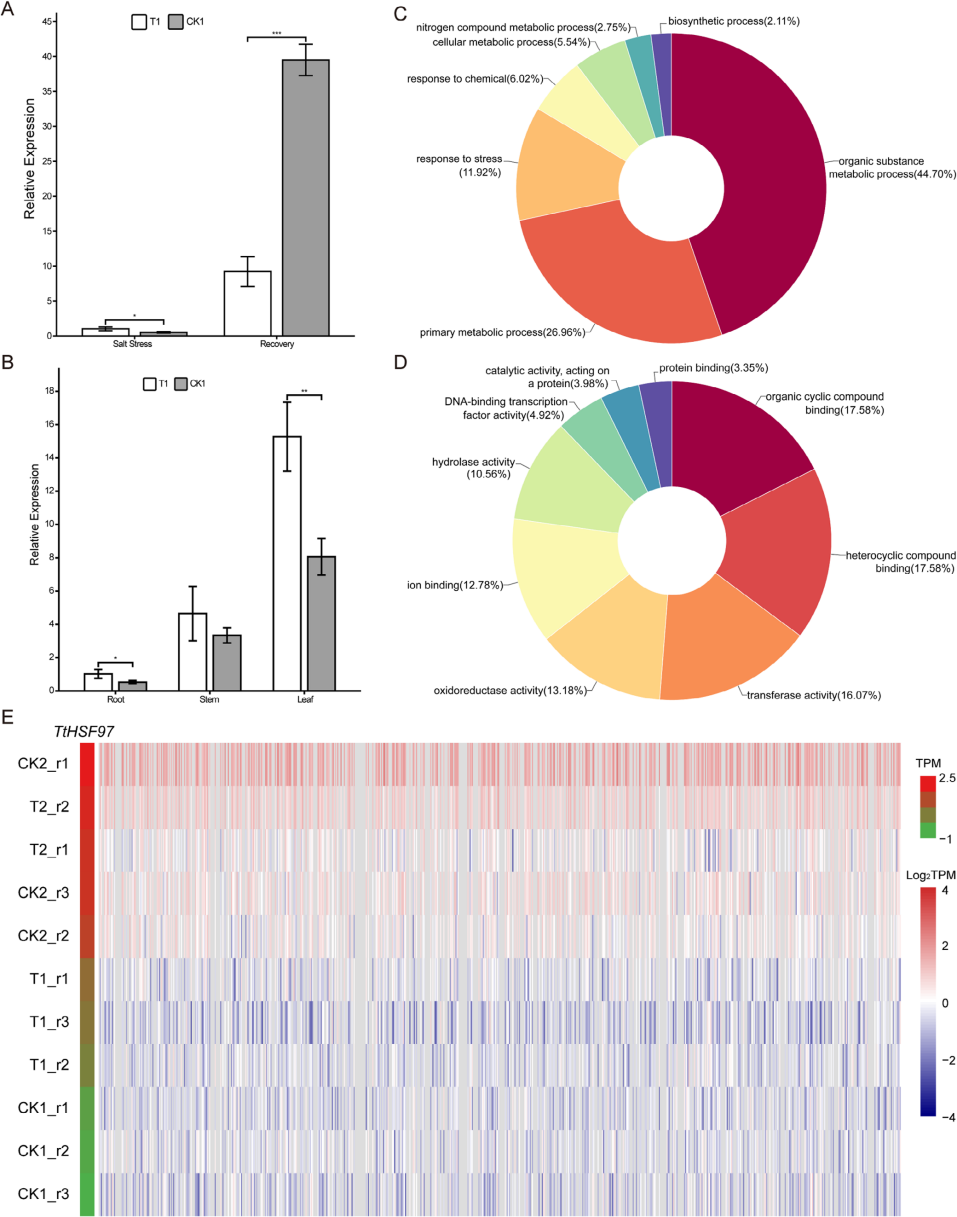
Expression patterns and correlation of *TtHSF97*. (A) Relative expression levels of *TtHSF97* in roots under salt stress and recovery conditions. Asterisks indicate significant differential expression vs. control (LSD test, *p* < 0.05). (B) Relative expression levels of *TtHSF97* in roots, stems, and leaves under salt stress (LSD test, *p* < 0.05). (C, D) Gene Ontology (GO) analysis of 733 genes positively correlated with *TtHSF97* expression, including biological process (BP; C) and molecular function (MF; D) categories. (E) A total of 733 genes with a high positive correlation (*R* > 0.9) to *TtHSF97* expression.

Previous studies demonstrated that overexpression of *AtHSFA2* (*AT2G26150*) confers enhanced tolerance to salt, osmotic, and hypoxic stresses. Similarly, *AtHSFA7b* activates ROS‐scavenging genes, reducing oxidative damage and improving salinity tolerance, which in turn improved the plant's salt tolerance. The *TtHSF97* gene (*Tel5E01T611500*) from the *Tritipyrum HSF* gene family, positioned on the same branch of the evolutionary tree with *AtHSFA2* and *AtHSFA7b* (Figure 2), was upregulated under salt stress and recovery treatment (Figure 5). To explore the spatial and temporal expression patterns of *TtHSF97*, qRT‐PCR analysis was conducted to assess its expression levels in roots under salt stress and recovery conditions, as well as in stems and leaves. Under salt stress, the expression level of *TtHSF97* in roots was significantly higher than that of the control, showing a 1.93‐fold increase (*p* < 0.05) (Figure 7A). These findings were in line with the transcriptome data mentioned earlier. Moreover, the relative expression level of *TtHSF97* was highest in the leaves of ‘Y1805’ under salt stress, followed by the stems and roots (Figure 7B). Under salt stress, the expression level of *TtHSF97* in the roots, stems, and leaves was higher than that in the control group. Specifically, the expression of *TtHSF97* in the roots increased by 0.78‐fold, which was significantly higher than that of the control (*p* < 0.05), while in the leaves, it increased by 1.12‐fold, showing a highly significant difference compared to the control (*p* < 0.01).

To investigate the biological processes of *TtHSF97* under salt stress and recovery treatment, a Pearson correlation analysis was conducted between *TtHSF97* and other genes within the transcriptome data. The analysis revealed that 733 genes (*R* > 0.9) exhibited a positive correlation with *TtHSF97* expression. This supports the potential role of TtHSF97 in regulating stress‐responsive genes. All of these genes showed high expression levels in the T_2_ samples (Figure 7E). Gene ontology (GO) analysis of these genes highlighted key BP and MF of GO annotation including organic substance metabolism, primary metabolism, stress response, chemical response, and cellular metabolic processes (Figure 7C,D). These findings collectively suggest that *TtHSF97* may play a role in mediating abiotic stress tolerance in plants.

**FIGURE 8:**
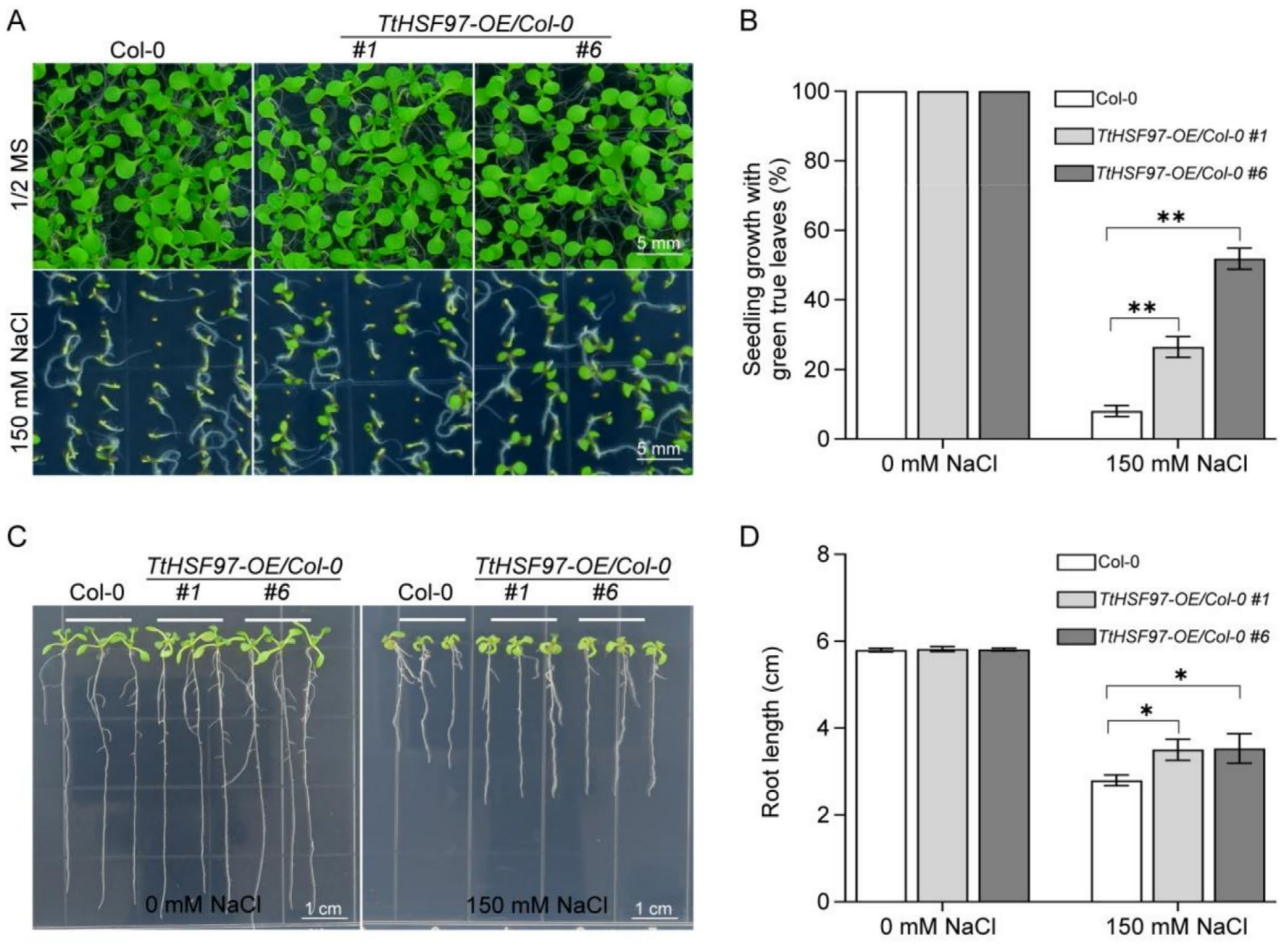
*TtHSF97* confers salt tolerance to *Arabidopsis* plants. (A) Phenotypic comparison of wild‐type (WT, Col‐0) and two homozygous *35S::TtHSF97* lines (#1, #6) under control (0 mM NaCl) and saline (150 mM NaCl) conditions. (B) Quantitative assessment of true leaf expansion rates after 12‐day exposure. Transgenic lines exhibited significantly higher expansion rates versus WT. **Denotes statistically significant differences between WT and transgenic lines (Student's *t*‐test, *p* < 0.01). Three independent biological experiments were performed. (C) The phenotypic comparison of different types of plant lines in culture medium under salinity or normal conditions. Seeds were plated on 1/2 MS or 1/2 MS medium containing 150 mM NaCl for 12 days to compare the root length. (D) The root length was compared. Three independent biological experiments were performed. *Significant difference compared with WT (*p* < 0.05).

Additionally, we investigated the function of WT and two different transgenic lines (OE#1 and OE#6), which had extremely significantly higher *TtHSF97* expression than WT under salt stress. Under control conditions (0 mM NaCl), all *Arabidopsis* plants, including both wild‐type (Col‐0) and *TtHSF97*‐overexpressing transgenic lines (#1 and #6), exhibited vigorous growth with no significant differences in green true leaf expansion rate or root length (Figure 8A,C). Following exposure to salt stress (150 mM NaCl), however, the transgenic lines overexpressing *TtHSF97* (#1 and #6) displayed significantly enhanced salt tolerance compared to WT. They maintained stronger growth vigor, exhibited reduced physiological damage, and showed a significantly higher green true leaf expansion rate. At this NaCl concentration, WT plants exhibited severe growth inhibition and were unable to fully expand their true leaves. Quantitative analysis revealed that the true leaf expansion rates for transgenic lines #1 and #6 were 39.0% and 53.7%, respectively, whereas the rate for WT plants was markedly lower at only 9.5% (Figure 8B; *p* < 0.001 for each transgenic line compared to WT, Student's *t*‐test). Furthermore, the root lengths of the overexpression lines were also significantly longer than those of WT under salt stress (Figure 8D).

### 3.7 Subcellular Localization of TtHSF97 Protein

**FIGURE 9:**
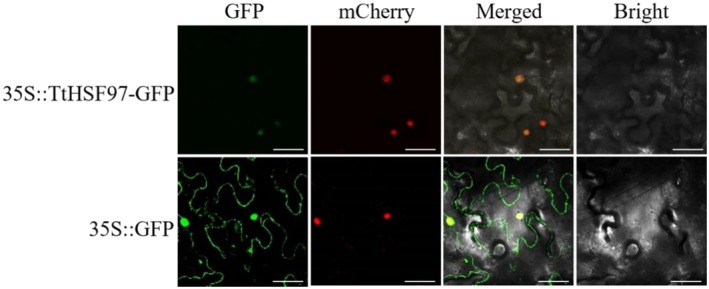
Subcellular localization of TtHSF97 by confocal imaging. Top: Diffuse GFP signal in *35S::GFP* control. Bottom: Exclusive nuclear accumulation of *35S::TtHSF97‐GFP*, co‐localizing with the mCherry‐tagged nuclear marker (Merge). 35S::TtHSF97‐GFP, a fusion protein: TtHSF97 protein was fused at the amino terminal of GFP. GFP, green fluorescent protein. Scale bar = 50 μm.

For subcellular localization analysis, a fusion construct *35S::TtHSF97‐GFP* was generated by ligating the *TtHSF97* CDS lacking its native stop codon to the N‐terminus of the fluorescent reporter gene GFP. Recombinant plasmids (*35S::TtHSF97‐GFP*) and the empty vector control (*35S::GFP*) were independently transformed into the abaxial epidermis of *N. benthamiana* leaves via *A. tumefaciens* ‐mediated transient transformation. Confocal microscopy images (Figure 9) revealed diffuse GFP fluorescence throughout the entire cell in control epidermal cells expressing *GFP*. In contrast, cells expressing the chimeric gene *TtHSF97‐GFP* exhibited exclusive nuclear GFP signals, which demonstrated precise colocalization with the mCherry fluorescence of a co‐expressed nuclear marker. This compartment‐specific localization confirms that TtHSF97 is an enriched protein, consistent with the canonical functional characteristics of transcription factors requiring nuclear access for DNA binding and transcriptional regulation.

## 4 Discussion

HSF, one of the largest plant transcription factor families, has been extensively studied, and their regulatory roles and molecular mechanisms in plant stress responses have become important research directions in the field of plant molecular biology. This study identified 103 *HSF* genes in *Tritipyrum*, substantially exceeding the numbers reported in *Arabidopsis* (21) (Nover et al. 2001), rice (25) (Guo et al. 2008), maize (30) (Lin et al. 2011), soybean (52) (Li et al. 2014), *Secale cereale* (31) (Li et al. 2021), and wheat (≥ 56) (Xue et al. 2014). This expansion likely reflects the combined effects of whole‐genome duplication (WGD), segmental duplication, and tandem duplication events, common drivers of gene family diversification in plants, and key mechanisms for environmental adaptation and stress resistance (Lallemand et al. 2020; Ren et al. 2018; Vanneste et al. 2014; Faraji et al. 2021; Rezaee et al. 2020; Heidari et al. 2021). Genomic studies on crops further confirm that such gene family expansion and duplication promote plant evolution and enhance viability across diverse environments (Shen and Yuan 2020; Li et al. 2021; Rehman et al. 2021). Collectively, these studies confirm that gene duplication events provide a critical molecular basis for the survival, reproduction, and evolution of crops in variable environments.

Notably, although salt‐tolerant species such as common wheat do not possess the highest number of *HSF* family members, their *HSF* members exhibit distinct characteristics in terms of functional differentiation at the subclass level and specialization of expression patterns, thereby forming an efficient multi‐dimensional stress regulatory network. Phylogenetic classification revealed that 103 *HSF* genes identified in *Tritipyrum* could be classified into three distinct groups, with Group A containing the highest proportion of *HSF* members. Comparative synteny analysis of the *HSF* genomes in *Tritipyrum*, *H. vulgare* , *O. sativa* , *S. cereale* , *Th. intermedium*, and maize demonstrated particularly strong conservation between *Tritipyrum* and *Th. intermedium*, supporting their close evolutionary relationship with traditional *Gramineae*. The genera *Tritipyrum* and *S. cereale* were found to have the fewest syntenic linkages. The identification of 1–13 *HSF* genes shared among at least three species in five syntenic relationships offers valuable insights into the evolutionary conservation of *HSF* genes across species. The functional differentiation of *HSFs* in such salt‐tolerant species is primarily manifested in three core subclasses. (1) The conservatively regulated *HSF* A2 subclass, serving as a “universal regulatory switch” for salt stress responses, is significantly upregulated by salt stress in diverse species including *Arabidopsis thaliana* , rice, and wheat. Its core function is to maintain intracellular protein homeostasis by activating the transcription of heat shock protein (HSP)‐encoding genes (e.g., *TaHsp*17 in wheat). In monocotyledonous plants, it further forms a coordinated regulatory pattern with the *HSF* C2 subclass, improving the precision of stress responses. (2) The salt tolerance‐specialized HSF A6 subclass exhibits a dual characteristic of “constitutive high expression + salt stress‐induced upregulation” in quinoa and wheat: its expression could increase sharply under salt stress (e.g., the expression level of the HSF A6e subclass in quinoa can reach 15 times the basal level). It is hypothesized to play a core role in establishing “stress memory” in embryonic and root tissues, enhancing the overall salt tolerance of plants by strengthening the stress response capacity of key organs. (3) The monocot‐specific HSF C2 subclass has an encoded protein that contains a unique AHA‐like activation domain (e.g., the LLLDGDFGNVSAFGPDAVDF). It can independently activate HSP transcription without relying on other HSFs, providing an additional regulatory layer for salt adaptation.

On this basis, salt‐tolerant species have also evolved a regulatory strategy of coordinated response among multiple HSF subclasses: Under salt stress, quinoa can synchronously induce the expression of members from five HSF subclasses (A2, A4, A6, B1, and C1), reducing the “failure risk” of stress responses through the redundancy of the regulatory network and improving adaptation stability (Giraldo Acosta et al. 2022). Common wheat, on the other hand, exhibits obvious organ‐specific division of labor—HSF A4 and A6 subclasses act as dominant regulatory factors in roots, with their transcriptional levels reaching more than 3 times those in leaf tissues; in contrast, the *HSF* B1 and C1 subclasses show stronger stress response activity in leaves, achieving efficient salt adaptation at the whole‐plant level through functional complementation between organs.

In summary, the expansion of the HSF gene family provides a genetic basis for functional differentiation, while functional specialization at the subclass level further drives the diversification of plant stress adaptation strategies. Future in‐depth analysis of the functions of crop‐specific HSFs (especially stress tolerance‐related subclasses) will provide an important theoretical basis for clarifying the molecular mechanisms of plant stress tolerance and guiding the genetic improvement of crop stress resistance, which is of great significance for breeding new crop varieties with high stress tolerance.

Salinity disrupts cellular homeostasis through osmotic and ionic imbalances (Tavakkoli et al. 2010). Unlike single functional genes, transcription factors (TFs), including HSFs, can simultaneously regulate multiple downstream genes involved in salt stress response. TFs such as bHLH, bZIP, MYC, NAC, WRKY, and HSF are associated with salt tolerance pathways (Golldack et al. 2011; Lan Thi Hoang et al. 2017; Meraj et al. 2020; Okushima et al. 2007). Numerous HSFs have been identified in response to various environmental stresses in species such as *Arabidopsis*, rice, maize, and wheat (Guo et al. 2016; Scharf et al. 2012; Xiang et al. 2013). The tissue‐specific expression of HSF genes, through regulation of transcriptional processes, can influence the growth and development of target tissues and organs (Guo et al. 2016; Scharf et al. 2012). In salt‐stressed *Arabidopsis*, the expression level of several *HSF* genes was upregulated, with *AtHSFA6a* showing a 146‐fold increase compared to the control (Miller and Mittler 2006), while *AtHSFA1* and *AtHSFA2* enhance seedling‐stage resistance to salt stress (Liu et al. 2011). Heterologous expression of wheat *TaHSFA2d* and rice *OsHSFA2e* genes in *Arabidopsis* improves salt tolerance, increasing germination rates and seedling stage chlorophyll content compared to wild‐type plants (Chauhan et al. 2013; Yokotani et al. 2007). Additionally, *OsHSFA2s*, *OsHSFA4s*, *OsHSFA7*, *OsHSFA9*, *OsHSFB2b*, and *FvHSFA2a* genes were upregulated by high salt stress, improving stress resistance to high salinity in rice and strawberry (Chauhan et al. 2013; Hu et al. 2015; Xiang et al. 2013). In this study, 84 *TtHSF* genes were identified in *Tritipyrum* root tissues, showing significant responses to salt stress induction. Among these, 41 showed constitutive expression, suggesting their involvement in regulating basic cellular processes, while 29 exhibited marked salt‐stress induction, suggesting their role in the salt stress adaptation. Expression profiling and qRT‐PCR analyses highlighted these 29 *TtHSFs* as potential key regulators of salt stress response in *Tritipyrum* root tissues. Further transgenic experiments will be conducted to elucidate the precise biological functions of these *TtHSFs*, with potential applications in genetic engineering to enhance crop stress resistance and other agronomic traits.

HSFs from different subfamilies can act as either positive or negative regulators of salt stress tolerance. For instance, overexpression of the wheat *TaHSFA2d* gene in *Arabidopsis* enhanced salt tolerance (0.3 mM NaCl), with transgenic lines exhibiting significantly higher seed germination rates and seedling chlorophyll content compared to wild‐type control plants. In contrast, seeds and seedlings of the *AtHSFA2* deletion mutant showed poorer growth than those of the wild type (Chauhan et al. 2013). Similarly, overexpression of *OsHSFA2e* in *Arabidopsis* enabled plants to survive up to 20% in an agar medium containing 0.125 mM NaCl, while all non‐transgenic plants died, indicating that *OsHSFA2e* enhances salt tolerance in *Arabidopsis* (Yokotani et al. 2007). In contrast, overexpression of *OsHSFB2b* in japonica rice treated with 0.2 mol·L^−1^ NaCl led to a marked decrease in salt tolerance, suggesting that *OsHSFB2b* acts as a negative regulator in the salt stress response and regulation (Xiang et al. 2013). These results underscore the functional divergence of *HSF* subfamilies in salt stress adaptation. *TtHSF97*, from *Tritipyrum*'s HSF family, shares the same evolutionary lineage. *AtHSFA2* in *Arabidopsis* emerged as a key candidate due to its significant upregulation under salt stresses and recovery. Overexpression of *AtHSFA2* can enhance the salt tolerance of transgenic *Arabidopsis thaliana* and simultaneously improve its tolerance to salt stress and osmotic stress (Zang et al. 2019). Studies have shown that the transcription level of *TaHsfA6f* in common wheat is significantly upregulated under multiple abiotic stress conditions, including salt stress. Overexpression of *TaHsfA6f* can enhance the tolerance of transgenic plants to heat, drought, and salt stresses, while increasing the plants' sensitivity to exogenous abscisic acid (ABA) and promoting ABA accumulation in vivo. This result indicates that *TaHsfA6f* enhances plant tolerance to multiple abiotic stresses by regulating the metabolic and signaling pathways of ABA (Bi et al. 2020).

Tissue‐specific profiling revealed highest relative expression of *TtHSF97* in leaves of *Tritipyrum* under salt stress, followed by stems and roots. High salt concentrations directly and severely damaged the root system. Under both salt stress and recovery, *TtHSF97* expression levels were significantly higher in the entire plant compared to the control. These findings align with our transcriptome data and previous reports (Chauhan et al. 2013). Consequently, *TtHSF97* was highly and sensitively expressed throughout the plant to assist in coping with salt stress. The genes closely related to *TtHSF97* are involved in processes such as metabolism, cellular functions, response to stimuli, and biological regulation. Functional validation via *Arabidopsis* heterologous overexpression of *TtHSF97* confirmed its positive regulatory role, enhancing transgenic lines salt stress tolerance, likely through early stress perception akin to *AtHSFA7b* (Zang et al. 2019). This mechanism may involve coordinating cellular defense mechanisms through activation of downstream stress‐responsive genes (e.g., SOS1, HSE‐containing targets). While this study establishes *TtHSF97*'s critical role, future studies will identify the specific cis‐regulatory motifs bound by *TtHSF97* via Chip‐seq or DAP‐seq, define its direct target genes to map its direct transcriptional network, and validate *TtHSF97* efficacy directly in triticale crops (e.g., wheat, *Tritipyrum*) via genetic engineering. In this study, we identified several genes significantly correlated with the expression of *TtHSF97*. For further investigation into the regulatory mechanisms of *TtHSF97*, we will utilize a yeast two‐hybrid system to screen for potential interacting target genes, particularly those involved in salt stress tolerance. This screening will help identify key genes that interact with *TtHSF97* and provide deep insights into how these genes contribute to salt tolerance. Subsequently, we plan to validate these interactions and explore their specific functions under salt stress, thus providing a more comprehensive understanding of the role of *TtHSF97* in stress adaptation.

Soil salinization has emerged as a critical factor, adversely affecting seed germination, seedling development, and crop yield, posing an increasingly serious challenge. *Th. elongatum*, a species closely related to *T. aestivum* , can thrive in salt concentrations comparable to those of seawater. *Tritipyrum*, a hybrid derived from intergeneric crossbreeding between *T. aestivum* and *Th. elongatum*, serves as a vital bridge for transferring salt tolerance genes from *Th. elongatum* into *T. aestivum* (Baker et al. 2020; McKenna et al. 2020). Enhancing salt tolerance in plants predominantly triggers stress‐responsive genes, whose products contribute to the repair of both primary and secondary stresses induced by salt.

Unlike individual functional genes, TFs, such as HSFs, have the capacity to regulate a group of downstream target genes, which, in turn, control physiological and biochemical processes responding to salt stress (Amirbakhtiar et al. 2019; van Zelm et al. 2020). In this study, we systematically characterized the *Tritipyrum HSF* family through phylogenetic, motif, and correlation analyses. RNA‐seq was employed as part of a transcriptomic analysis under salt stress identified *TtHSF97 as a key candidate, homologous to Arabidopsis AtHSFA2*. Subcellular localization confirmed its nuclear targeting (Figure 9), consistent with its role as a transcriptional regulator. Heterologous expression of *TtHSF97* in *Arabidopsis* validated its salt‐responsive function, with transgenic lines exhibiting 38%–54% leaf expansion than WT (9.5%) under 150 mM NaCl (Figure 8B,C). These findings position *TtHSF97* as a promising regulator of salinity resilience. In particular, our overexpression experiments in *Arabidopsis* have confirmed that the constitutive expression of *TtHSF97* can significantly enhance the salt tolerance of transgenic plants, including higher survival rates, faster leaf expansion rates, and more developed root systems architecture. This makes it a highly promising target for genetic engineering. By introducing *TtHSF97* or its allelic variants into major food crops such as common wheat through molecular marker‐assisted selection (MAS) or transgenic technology, it is expected to develop new varieties with stronger adaptability to saline‐alkali soils, thereby directly addressing the threat of global soil salinization to agricultural production. Studies have confirmed that salt‐tolerant plants can maintain high protein content and nutritional stability under salt stress: although some quinoa genotypes show reduced yield due to increased salt concentration, they still retain high seed protein content (Hussain et al. 2020); salt‐tolerant rice genotypes exhibit significantly better yield and appearance quality than salt‐sensitive ones (Li et al. 2023). This may stem from the close association between salt tolerance and plant physiological metabolism, and salt tolerance can also positively regulate nutritional value (Ullah et al. 2019; Duan et al. 2023; Ben‐Amar et al. 2022). Specific genes (e.g., OsGrx_C7 in rice) can enhance salt tolerance and promote biomass accumulation and root development by regulating metabolic pathways under salt stress (Verma et al. 2021; Zang et al. 2025; Zhang et al. 2020; Ali et al. 2025). In conclusion, improving plant salt tolerance not only helps plants survive under salt stress but also can enhance food crop quality and nutritional stability through multiple physiological and molecular mechanisms. Plant response to salt stress is highly complex, involving numerous genes and pathways. The deployment of *HSF*‐based genetic engineering promises to facilitate the cultivation of salt‐tolerant crops, driven by the discovery and widespread use of candidate transcription factor genes linked to salt tolerance, along with the ongoing advancement in understanding the transcription factor‐mediated salt tolerance mechanism.

## 5 Conclusions

This study provides a comprehensive analysis of the *HSF* gene family in *Tritipyrum*. The motif compositions of the 103 full‐length *TtHSF* genes within the same groups and subgroups exhibit striking similarity. Synteny analysis and phylogenetic comparisons of *HSF* genes across multiple plant species revealed the evolutionary features of *HSF* genes in *Tritipyrum*. Based on their expression patterns in various tissues and their response to salt stress and recovery treatments, 29 *TtHSF* genes are pivotal in the salt stress response of *Tritipyrum*. *TtHSF97*, a top‐priority salt‐responsive gene among 29 *TtHSFs*, exhibits nuclear localization, consistent with its function as a transcriptional regulator. Heterologous expression of *TtHSF97* in *Arabidopsis* significantly enhances salinity tolerance, indicating *TtHSF97* could serve as a potential target gene for enhancing salt tolerance in wheat through biotechnological or molecular breeding approaches. These findings provide valuable insights into the biological functions of specific *HSF* genes in *Tritipyrum*.

## Author Contributions

**Xianjiao Qin:** data curation (equal), formal analysis (equal), investigation (equal), writing – original draft (equal). **Wenzhen Li:** investigation (equal), writing – original draft (equal). **Ruoruo Wang:** writing – review and editing (equal). **Jianxia Xu:** data curation (equal), investigation (equal). **Yanqing Ding:** investigation (equal), visualization (equal). **Kuiyin Li:** data curation (equal), funding acquisition (equal), writing – review and editing (equal).

## Conflicts of Interest

The authors declare no conflicts of interest.

## Supporting information


**Figure S1:** Conserved motif distribution of HSF proteins in *Tritipyrum*.
**Figure S2:** Genetic distance distribution among HSF subfamilies.

**Table S1:** Systematic renaming of HSF genes based on chromosomal positions in the *Tritipyrum* genome.

**Table S2:** Forward and reverse primers used for qRT‐RCR validation of TtHSF gene expression.

## Data Availability

The data that support the findings of this study are available from the corresponding author upon reasonable request.

## References

[fsn371418-bib-0001] Albihlal, W. S. , I. Obomighie , T. Blein , et al. 2018. “ *Arabidopsis* Heat Shock Transcription Factor A1b Regulates Multiple Developmental Genes Under Benign and Stress Conditions.” Journal of Experimental Botany 69, no. 11: 2847–2862. 10.1093/jxb/ery142. 29697803 PMC5961379

[fsn371418-bib-0002] Ali, S. , M. Khan , and Y. S. Moon . 2025. “Synergistic Effect of *Serratia fonticola* and *Pseudomonas koreensis* on Mitigating Salt Stress in *Cucumis sativus* L.” Current Issues in Molecular Biology 47, no. 3: 194. 10.3390/cimb47030194. 40136448 PMC11941737

[fsn371418-bib-0003] Amirbakhtiar, N. , A. Ismaili , M. R. Ghaffari , F. Nazarian Firouzabadi , and Z. S. Shobbar . 2019. “Transcriptome Response of Roots to Salt Stress in a Salinity‐Tolerant Bread Wheat Cultivar.” PLoS One 14: e0213305. 10.1371/journal.pone.0213305. 30875373 PMC6420002

[fsn371418-bib-0005] Baker, L. , S. Grewal , C. Y. Yang , et al. 2020. “Exploiting the Genome of *Thinopyrum elongatum* to Expand the Gene Pool of Hexaploid Wheat.” Theoretical and Applied Genetics 133: 2213–2226. 10.1007/s00122-020-03583-3. 32313991 PMC7311493

[fsn371418-bib-0006] Bakery, A. , S. Vraggalas , B. Shalha , H. Chauhan , M. Benhamed , and S. Fragkostefanakis . 2024. “Heat Stress Transcription Factors as the Central Molecular Rheostat to Optimize Plant Survival and Recovery From Heat Stress.” New Phytologist 244, no. 1: 51–64. 10.1111/nph.20017. 39061112

[fsn371418-bib-0007] Ben‐Amar, A. , S. Daldoul , D. Allel , T. Wetzel , and A. Mliki . 2022. “Ectopic Expression of a Grapevine Alkaline α‐Galactosidase Seed Imbibition Protein VvSIP Enhanced Salinity Tolerance in Transgenic Tobacco Plants.” Functional & Integrative Genomics 23, no. 1: 12. 10.1007/s10142-022-00945-6. 36547729

[fsn371418-bib-0008] Bi, H. , J. Miao , J. He , et al. 2022. “Characterization of the Wheat Heat Shock Factor TaHsfA2e‐5D Conferring Heat and Drought Tolerance in *Arabidopsis* .” International Journal of Molecular Sciences 23, no. 5: 2784. 10.3390/ijms23052784. 35269925 PMC8911409

[fsn371418-bib-0009] Bi, H. , Y. Zhao , H. Li , and W. Liu . 2020. “Wheat Heat Shock Factor TaHsfA6f Increases ABA Levels and Enhances Tolerance to Multiple Abiotic Stresses in Transgenic Plants.” International Journal of Molecular Sciences 21, no. 9: 3121. 10.3390/ijms21093121. 32354160 PMC7247712

[fsn371418-bib-0010] Buchfink, B. , C. Xie , and D. H. Huson . 2015. “Fast and Sensitive Protein Alignment Using DIAMOND.” Nature Methods 12: 59–60. 10.1038/nmeth.3176. 25402007

[fsn371418-bib-0011] Chauhan, H. , N. Khurana , P. Agarwal , J. P. Khurana , and P. Khurana . 2013. “A Seed Preferential Heat Shock Transcription Factor From Wheat Provides Abiotic Stress Tolerance and Yield Enhancement in Transgenic *Arabidopsis* Under Heat Stress Environment.” PLoS One 8: e79577. 10.1371/journal.pone.0079577. 24265778 PMC3827158

[fsn371418-bib-0012] Döring, P. , E. Treuter , C. Kistner , R. Lyck , A. Chen , and L. Nover . 2000. “The Role of AHA Motifs in the Activator Function of Tomato Heat Stress Transcription Factors HsfA1 and HsfA2.” Plant Cell 12, no. 2: 265–278. 10.1105/tpc.12.2.265. 10662862 PMC139763

[fsn371418-bib-0013] Duan, H. , R. J. Tiika , F. Tian , et al. 2023. “Metabolomics Analysis Unveils Important Changes Involved in the Salt Tolerance of *Salicornia europaea* .” Frontiers in Plant Science 13: 1097076. 10.3389/fpls.2022.1097076. 36743536 PMC9896792

[fsn371418-bib-0014] Faraji, S. , P. Heidari , H. Amouei , E. Filiz , Abdullah , and P. Poczai . 2021. “Investigation and Computational Analysis of the Sulfotransferase (SOT) Gene Family in Potato ( *Solanum tuberosum* ): Insights Into Sulfur Adjustment for Proper Development and Stimuli Responses.” Plants 10: 2597. 10.3390/plants10122597. 34961068 PMC8707064

[fsn371418-bib-0015] Giraldo Acosta, M. , A. Cano , J. Hernández‐Ruiz , and M. B. Arnao . 2022. “Melatonin as a Possible Natural Safener in Crops.” Plants (Basel) 11, no. 7: 890. 10.3390/plants11070890. 35406870 PMC9003551

[fsn371418-bib-0016] Golldack, D. , I. Lüking , and O. Yang . 2011. “Plant Tolerance to Drought and Salinity: Stress Regulating Transcription Factors and Their Functional Significance in the Cellular Transcriptional Network.” Plant Cell Reports 30: 1383–1391. 10.1007/s00299-011-1068-0. 21476089

[fsn371418-bib-0017] Guo, J. , J. Wu , Q. Ji , et al. 2008. “Genome‐Wide Analysis of Heat Shock Transcription Factor Families in Rice and *Arabidopsis* .” Journal of Genetics and Genomics 35: 105–118. 10.1016/S1673-8527(08)60016-8. 18407058

[fsn371418-bib-0018] Guo, M. , J. H. Liu , X. Ma , D. X. Luo , Z. H. Gong , and M. H. Lu . 2016. “The Plant Heat Stress Transcription Factors (HSFs): Structure, Regulation, and Function in Response to Abiotic Stresses.” Frontiers in Plant Science 7: 114. 10.3389/fpls.2016.00114. 26904076 PMC4746267

[fsn371418-bib-0019] Haj‐Amor, Z. , T. Araya , D. G. Kim , et al. 2022. “Soil Salinity and Its Associated Effects on Soil Microorganisms, Greenhouse Gas Emissions, Crop Yield, Biodiversity and Desertification: A Review.” Science of the Total Environment 843: 156946. 10.1016/j.scitotenv.2022.156946. 35768029

[fsn371418-bib-0020] Heidari, P. , S. Faraji , and P. Poczai . 2021. “Magnesium Transporter Gene Family: Genome‐Wide Identification and Characterization in *Theobroma cacao*, *Corchorus capsularis*, and *Gossypium hirsutum* of Family Malvaceae.” Agronomy 11: 1651. 10.3390/agronomy11081651.

[fsn371418-bib-0021] Hu, Y. , Y. T. Han , W. Wei , et al. 2015. “Identification, Isolation, and Expression Analysis of Heat Shock Transcription Factors in the Diploid Woodland Strawberry *Fragaria vesca* .” Frontiers in Plant Science 6: 736. 10.3389/fpls.2015.00736. 26442049 PMC4569975

[fsn371418-bib-0022] Hussain, M. I. , A. Muscolo , M. Ahmed , et al. 2020. “Yield and Quality Traits and Interrelationship With Yield Stability in Quinoa (*Chenopodium quinoa* Willd.) Genotypes Under Saline Marginal Environment.” Plants (Basel) 9, no. 12: 1763. 10.3390/plants9121763. 33322139 PMC7764209

[fsn371418-bib-0023] Kumar, S. , S. R. Jacob , R. R. Mir , et al. 2022. “Indian Wheat Genomics Initiative for Harnessing the Potential of Wheat Germplasm Resources for Breeding Disease‐Resistant, Nutrient‐Dense, and Climate‐Resilient Cultivars.” Frontiers in Genetics 13: 834366. 10.3389/fgene.2022.834366. 35846116 PMC9277310

[fsn371418-bib-0024] Lallemand, T. , M. Leduc , C. Landès , C. Rizzon , and E. Lerat . 2020. “An Overview of Duplicated Gene Detection Methods: Why the Duplication Mechanism Has to Be Accounted for in Their Choice.” Genes 11, no. 9: 1046. 10.3390/genes11091046. 32899740 PMC7565063

[fsn371418-bib-0025] Lan Thi Hoang, X. , N. H. Du Nhi , N. Binh Anh Thu , et al. 2017. “Transcription Factors and Their Roles in Signal Transduction in Plants Under Abiotic Stresses.” Current Genomics 18: 483–497. 10.2174/1389202918666170227150057. 29204078 PMC5684650

[fsn371418-bib-0026] Letunic, I. , T. Doerks , and P. Bork . 2012. “SMART 7: Recent Updates to the Protein Domain Annotation Resource.” Nucleic Acids Research 40, no. D1: D302–D305. 10.1093/nar/gkr931. 22053084 PMC3245027

[fsn371418-bib-0027] Li, J. , L. Pu , M. Han , M. Zhu , R. Zhang , and Y. Xiang . 2014. “Soil Salinization Research in China: Advances and Prospects.” Journal of Geographical Sciences 24: 943–960. 10.1007/s11442-014-1130-2.

[fsn371418-bib-0028] Li, K. , L. Duan , Y. Zhang , et al. 2021. “Genome‐Wide Identification and Expression Profile Analysis of Trihelix Transcription Factor Family Genes in Response to Abiotic Stress in Sorghum [ *Sorghum bicolor* (L.) Moench].” BMC Genomics 22, no. 1: 738. 10.1186/s12864-021-08000-7. 34649496 PMC8515681

[fsn371418-bib-0030] Li, Z. J. , L. L. Zhang , A. Wang , et al. 2013. “Ectopic Overexpression of SlHsfA3, a Heat Stress Transcription Factor From Tomato, Confers Increased Thermotolerance and Salt Hypersensitivity in Germination in Transgenic *Arabidopsis* .” PLoS One 8, no. 1: e54880. 10.1371/journal.pone.0054880. 23349984 PMC3551807

[fsn371418-bib-0029] Li, Z. , T. Zhou , K. Zhu , et al. 2023. “Effects of Salt Stress on Grain Yield and Quality Parameters in Rice Cultivars With Differing Salt Tolerance.” Plants (Basel) 12, no. 18: 3243. 10.3390/plants12183243. 37765407 PMC10538069

[fsn371418-bib-0031] Liang, W. , X. Ma , P. Wan , and L. Liu . 2018. “Plant Salt‐Tolerance Mechanism: A Review.” Biochemical and Biophysical Research Communications 495, no. 1: 286–291. 10.1016/j.bbrc.2017.11.043. 29128358

[fsn371418-bib-0032] Liao, Y. , G. K. Smyth , and W. Shi . 2014. “featureCounts: An Efficient General Purpose Program for Assigning Sequence Reads to Genomic Features.” Bioinformatics 30: 923–930. 10.1093/bioinformatics/btt656. 24227677

[fsn371418-bib-0033] Lin, Y. X. , H. Y. Jiang , Z. X. Chu , X. L. Tang , S. W. Zhu , and B. J. Cheng . 2011. “Genome‐Wide Identification, Classification and Analysis of Heat Shock Transcription Factor Family in Maize.” BMC Genomics 12: 76. 10.1186/1471-2164-12-76. 21272351 PMC3039612

[fsn371418-bib-0034] Litalien, A. , and B. Zeeb . 2020. “Curing the Earth: A Review of Anthropogenic Soil Salinization and Plant‐Based Strategies for Sustainable Mitigation.” Science of the Total Environment 698: 134235. 10.1016/j.scitotenv.2019.134235. 31783465

[fsn371418-bib-0035] Liu, H. C. , H. T. Liao , and Y. Y. Charng . 2011. “The Role of Class A1 Heat Shock Factors (HSFA1s) in Response to Heat and Other Stresses in *Arabidopsis* .” Plant, Cell & Environment 34: 738–751. 10.1111/j.1365-3040.2011.02278.x. 21241330

[fsn371418-bib-0077] Liu, P. , J. Ma , D. Zhang , et al. 2014. “Genome‐wide analysis of the WRKY transcription factors in Aegilops tauschii.” Genetics and Molecular Research 13, no. 4: 9903–9915. 10.1159/000370172 25592959

[fsn371418-bib-0037] Mao, J. , Y. C. Zhang , Y. Sang , Q.‐H. Li , and H.‐Q. Yang . 2005. “A Role for *Arabidopsis* Cryptochromes and COP1 in the Regulation of Stomatal Opening.” Proceedings of the National Academy of Sciences of the United States of America 102: 12270–12275. 10.1073/pnas.0501011102. 16093319 PMC1189306

[fsn371418-bib-0038] Mayer, K. F. X. , J. Rogers , J. Doležel , et al. 2014. “A Chromosome‐Based Draft Sequence of the Hexaploid Bread Wheat (*Triticum aestivum*) Genome.” Science 345, no. 6194: 1251788. 10.1126/science.1251788. 25035500

[fsn371418-bib-0039] McKenna, T. P. , L. Koziol , J. D. Bever , T. E. Crews , and B. A. Sikes . 2020. “Abiotic and Biotic Context Dependency of Perennial Crop Yield.” PLoS One 15: e0234546. 10.1371/journal.pone.0234546. 32589642 PMC7319328

[fsn371418-bib-0040] Meraj, T. A. , J. Fu , M. A. Raza , et al. 2020. “Transcriptional Factors Regulate Plant Stress Responses Through Mediating Secondary Metabolism.” Genes 11, no. 4: 346. 10.3390/genes11040346. 32218164 PMC7230336

[fsn371418-bib-0041] Miller, G. A. D. , and R. O. N. Mittler . 2006. “Could Heat Shock Transcription Factors Function as Hydrogen Peroxide Sensors in Plants?” Annals of Botany 98, no. 2: 279–288. 10.1093/aob/mcl107. 16740587 PMC2803459

[fsn371418-bib-0042] Muleta, T. 2024. “Soil Salinity Causes, Effects, and Its Managements.” Biomedical Journal of Scientific & Technical Research 59, no. 2: 51373–51739. 10.26717/BJSTR.2024.59.009270.

[fsn371418-bib-0043] Nishizawa‐Yokoi, A. , R. Nosaka , H. Hayashi , et al. 2011. “HsfA1d and HsfA1e Involved in the Transcriptional Regulation of HsfA2 Function as Key Regulators for the Hsf Signaling Network in Response to Environmental Stress.” Plant and Cell Physiology 52, no. 5: 933–945. 10.1093/pcp/pcr045. 21471117

[fsn371418-bib-0044] Nover, L. , K. Bharti , P. Döring , S. K. Mishra , A. Ganguli , and K. D. Scharf . 2001. “ *Arabidopsis* and the Heat Stress Transcription Factor World: How Many Heat Stress Transcription Factors Do We Need?” Cell Stress & Chaperones 6, no. 3: 177. 11599559 10.1379/1466-1268(2001)006<0177:aathst>2.0.co;2 PMC434399

[fsn371418-bib-0045] Ogawa, D. , K. Yamaguchi , and T. Nishiuchi . 2007. “High‐Level Overexpression of the *Arabidopsis* HsfA2 Gene Confers Not Only Increased Themotolerance but Also Salt/Osmotic Stress Tolerance and Enhanced Callus Growth.” Journal of Experimental Botany 58, no. 12: 3373–3383. 10.1093/jxb/erm184. 17890230

[fsn371418-bib-0046] Okushima, Y. , H. Fukaki , M. Onoda , A. Theologis , and M. Tasaka . 2007. “ARF7 and ARF19 Regulate Lateral Root Formation via Direct Activation ofLBD/ASLGenes in *Arabidopsis* .” Plant Cell 19, no. 1: 118–130. 10.1105/tpc.106.047761. 17259263 PMC1820965

[fsn371418-bib-0047] Rehman, S. U. , G. Qanmber , M. H. N. Tahir , et al. 2021. “Characterization of Vascular Plant One‐Zinc Finger (VOZ) in Soybean (*Glycine max* and *Glycine soja* ) and Their Expression Analyses Under Drought Condition.” PLoS One 16, no. 7: e0253836. 10.1371/journal.pone.0253836. 34214130 PMC8253436

[fsn371418-bib-0048] Ren, R. , H. Wang , C. Guo , et al. 2018. “Widespread Whole Genome Duplications Contribute to Genome Complexity and Species Diversity in Angiosperms.” Molecular Plant 11, no. 3: 414–428. 10.1016/j.molp.2018.01.002. 29317285

[fsn371418-bib-0049] Rezaee, S. , M. Ahmadizadeh , and P. Heidari . 2020. “Genome‐Wide Characterization, Expression Profiling, and Post‐Transcriptional Study of GASA Gene Family.” Gene Reports 20: 100795. 10.1016/j.genrep.2020.100795.

[fsn371418-bib-0050] Scharf, K. D. , T. Berberich , I. Ebersberger , and L. Nover . 2012. “The Plant Heat Stress Transcription Factor (Hsf) Family: Structure, Function and Evolution.” Biochimica et Biophysica Acta (BBA) – Gene Regulatory Mechanisms 1819, no. 2: 104–119. 10.1016/j.bbagrm.2011.10.002. 22033015

[fsn371418-bib-0051] Scharf, K. D. , S. Rose , W. Zott , F. Schöffl , L. Nover , and F. Schöff . 1990. “Three Tomato Genes Code for Heat Stress Transcription Factors With a Region of Remarkable Homology to the DNA‐Binding Domain of the Yeast HSF.” EMBO Journal 9, no. 13: 4495–4501. 10.1002/j.1460-2075.1990.tb07900.x. 2148291 PMC552242

[fsn371418-bib-0052] Segarra‐Medina, C. , A. Gómez‐Cadenas , and S. I. Zandalinas . 2025. “Physiological, Molecular, and Metabolic Adaptations of Plants to Combined Salinity and High Irradiance Stress.” Physiologia Plantarum 177, no. 2: e70164. 10.1111/ppl.70164. 40128164

[fsn371418-bib-0053] Shen, C. , and J. Yuan . 2020. “Genome‐Wide Characterization and Expression Analysis of the Heat Shock Transcription Factor Family in Pumpkin ( *Cucurbita moschata* ).” BMC Plant Biology 20, no. 1: 471. 10.1186/s12870-020-02683-y. 33054710 PMC7557022

[fsn371418-bib-0054] Singh, P. , K. K. Choudhary , N. Chaudhary , et al. 2022. “Salt Stress Resilience in Plants Mediated Through Osmolyte Accumulation and Its Crosstalk Mechanism With Phytohormones.” Frontiers in Plant Science 13: 1006617. 10.3389/fpls.2022.1006617. 36237504 PMC9552866

[fsn371418-bib-0055] Tang, X. , X. Mu , H. Shao , H. Wang , and M. Brestic . 2014. “Global Plant‐Responding Mechanisms to Salt Stress: Physiological and Molecular Levels and Implications in Biotechnology.” Critical Reviews in Biotechnology 35, no. 4: 425–437. 10.3109/07388551.2014.889080. 24738851

[fsn371418-bib-0056] Tavakkoli, E. , P. Rengasamy , and G. K. McDonald . 2010. “High Concentrations of Na+ and cl– Ions in Soil Solution Have Simultaneous Detrimental Effects on Growth of Faba Bean Under Salinity Stress.” Journal of Experimental Botany 61, no. 15: 4449–4459. 10.1093/jxb/erq251. 20713463 PMC2955754

[fsn371418-bib-0057] Tian, H. , Y. Mu , S. Yang , et al. 2024. “ATAC Sequencing and Transcriptomics Reveal the Impact of Chromatin Accessibility on Gene Expression in *Tritipyrum* Under Salt‐Stress Conditions.” Environmental and Experimental Botany 228: 106014. 10.1016/j.envexpbot.2024.106014.

[fsn371418-bib-0058] Treuter, E. , L. Nover , K. Ohme , and K. D. Scharf . 1993. “Promoter Specificity and Deletion Analysis of Three Heat Stress Transcription Factors of Tomato.” Molecular and General Genetics MGG 240, no. 1: 113–125. 10.1007/bf00276890. 8341257

[fsn371418-bib-0060] Ullah, A. , M. Li , J. Noor , A. Tariq , Y. Liu , and L. Shi . 2019. “Effects of Salinity on Photosynthetic Traits, Ion Homeostasis and Nitrogen Metabolism in Wild and Cultivated Soybean.” PeerJ 7: e8191. 10.7717/peerj.8191. 31844583 PMC6907091

[fsn371418-bib-0061] van Zelm, E. , Y. Zhang , and C. Testerink . 2020. “Salt Tolerance Mechanisms of Plants.” Annual Review of Plant Biology 71, no. 1: 403–433. 10.1146/annurev-arplant-050718-100005. 32167791

[fsn371418-bib-0062] Vanneste, K. , G. Baele , S. Maere , and Y. de Van Peer . 2014. “Analysis of 41 Plant Genomes Supports a Wave of Successful Genome Duplications in Association With the Cretaceous–Paleogene Boundary.” Genome Research 24, no. 8: 1334–1347. 10.1101/gr.168997.113. 24835588 PMC4120086

[fsn371418-bib-0063] Verma, P. K. , S. Verma , R. D. Tripathi , N. Pandey , and D. Chakrabarty . 2021. “CC‐Type Glutaredoxin, OsGrx_C7 Plays a Crucial Role in Enhancing Protection Against Salt Stress in Rice.” Journal of Biotechnology 329: 192–203. 10.1016/j.jbiotec.2021.02.008. 33610657

[fsn371418-bib-0064] von Koskull‐Döring, P. , K.‐D. Scharf , and L. Nover . 2007. “The Diversity of Plant Heat Stress Transcription Factors.” Trends in Plant Science 12, no. 10: 452–457. 10.1016/j.tplants.2007.08.014. 17826296

[fsn371418-bib-0065] Wang, H. , S. Sun , W. Ge , et al. 2020. “Horizontal Gene Transfer of Fhb7 From Fungus Underlies Fusarium Head Blight Resistance in Wheat.” Science 368, no. 6493: eaba5435. 10.1126/science.aba5435. 32273397

[fsn371418-bib-0066] Wang, X. , Z. Chen , and N. Sui . 2024. “Sensitivity and Responses of Chloroplasts to Salt Stress in Plants.” Frontiers in Plant Science 15: 1374086. 10.3389/fpls.2024.1374086. 38693929 PMC11061501

[fsn371418-bib-0067] Weigel, D. , and J. Glazebrook . 2006. “Transformation of Agrobacterium Using the Freeze‐Thaw Method.” Cold Spring Harbor Protocols 2006, no. 7: 4666. 10.1101/pdb.prot4666. 22484682

[fsn371418-bib-0068] Xiang, J. , J. Ran , J. Zou , et al. 2013. “Heat Shock Factor OsHsfB2b Negatively Regulates Drought and Salt Tolerance in Rice.” Plant Cell Reports 32, no. 11: 1795–1806. 10.1007/s00299-013-1492-4. 23949687

[fsn371418-bib-0069] Xu, Y. , Y. Zhao , H. Duan , N. Sui , F. Yuan , and J. Song . 2017. “Transcriptomic Profiling of Genes in Matured Dimorphic Seeds of Euhalophyte Suaeda Salsa.” BMC Genomics 18, no. 1: 727. 10.1186/s12864-017-4104-9. 28903734 PMC5598043

[fsn371418-bib-0070] Xue, G.‐P. , S. Sadat , J. Drenth , and C. L. McIntyre . 2014. “The Heat Shock Factor Family From *Triticum aestivum* in Response to Heat and Other Major Abiotic Stresses and Their Role in Regulation of Heat Shock Protein Genes.” Journal of Experimental Botany 65, no. 2: 539–557. 10.1093/jxb/ert399. 24323502 PMC3904712

[fsn371418-bib-0071] Yao, Q. , P. Pan , X. Zheng , G. Zhou , and J. Zhang . 2025. “ST‐YOLO: A Deep Learning Based Intelligent Identification Model for Salt Tolerance of Wild Rice Seedlings.” Frontiers in Plant Science 16: 1595386. 10.3389/fpls.2025.1595386. 40530276 PMC12171364

[fsn371418-bib-0072] Yokotani, N. , T. Ichikawa , Y. Kondou , et al. 2007. “Expression of Rice Heat Stress Transcription Factor OsHsfA2e Enhances Tolerance to Environmental Stresses in Transgenic *Arabidopsis* .” Planta 227, no. 5: 957–967. 10.1007/s00425-007-0670-4. 18064488

[fsn371418-bib-0073] Yu, Z. , X. Duan , L. Luo , S. Dai , Z. Ding , and G. Xia . 2020. “How Plant Hormones Mediate Salt Stress Responses.” Trends in Plant Science 25, no. 11: 1117–1130. 10.1016/j.tplants.2020.06.008. 32675014

[fsn371418-bib-0074] Zang, D. , Y. Duan , H. Zhao , et al. 2025. “Transcriptome and Metabolome Analyses of the Salt Stress Response Mechanism in *Lonicera caerulea* .” Biology 14, no. 6: 641. 10.3390/biology14060641. 40563892 PMC12189683

[fsn371418-bib-0075] Zang, D. , J. Wang , X. Zhang , Z. Liu , and Y. Wang . 2019. “ *Arabidopsis* Heat Shock Transcription Factor HSFA7b Positively Mediates Salt Stress Tolerance by Binding to an E‐Box‐Like Motif to Regulate Gene Expression.” Journal of Experimental Botany 70, no. 19: 5355–5374. 10.1093/jxb/erz261. 31145794 PMC6793466

[fsn371418-bib-0076] Zhang, S. , X. Li , S. Fan , L. Zhou , and Y. Wang . 2020. “Overexpression of HcSCL13, a Halostachys Caspica GRAS Transcription Factor, Enhances Plant Growth and Salt Stress Tolerance in Transgenic *Arabidopsis* .” Plant Physiology and Biochemistry 151: 243–254. 10.1016/j.plaphy.2020.03.020. 32240936

