# Supplementary material for: Genome‐Wide Analysis of Tritipyrum HSFs and Functional Characterization of TtHSF97 for Salt Tolerance
Source: Food Sci Nutr. 2026 Jan 10;14(1):e71418. doi: 10.1002/fsn3.71418 (PMC12789894; doi:10.1002/fsn3.71418)
Supplement: Supplementary file 1 — Figure S1: Conserved motif distribution of HSF proteins in Tritipyrum. Figure S2: Genetic distance distribution among HSF subfamilies. [file FSN3-14-e71418-s001.docx]

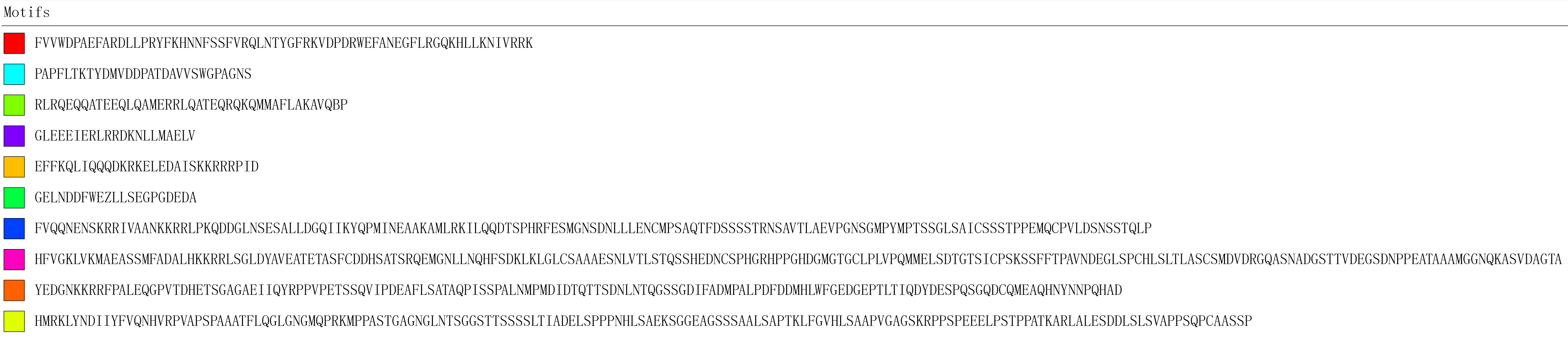


**Fig. S1. Conserved motif distribution of HSF proteins in *Tritipyrum*.**

Phylogenetic analysis of HSF proteins from *Tritipyrum* was performed using the Neighbor-Joining (NJ) method with 1000 bootstrap replicates based on full-length amino acid sequences. Conserved motifs of TtHSF proteins were identified using the MEME suite. Different colors represent distinct conserved motifs, and motif composition patterns are shown alongside the phylogenetic tree. Proteins belonging to the same subgroup exhibit similar motif architectures, indicating functional conservation within HSF subfamilies.


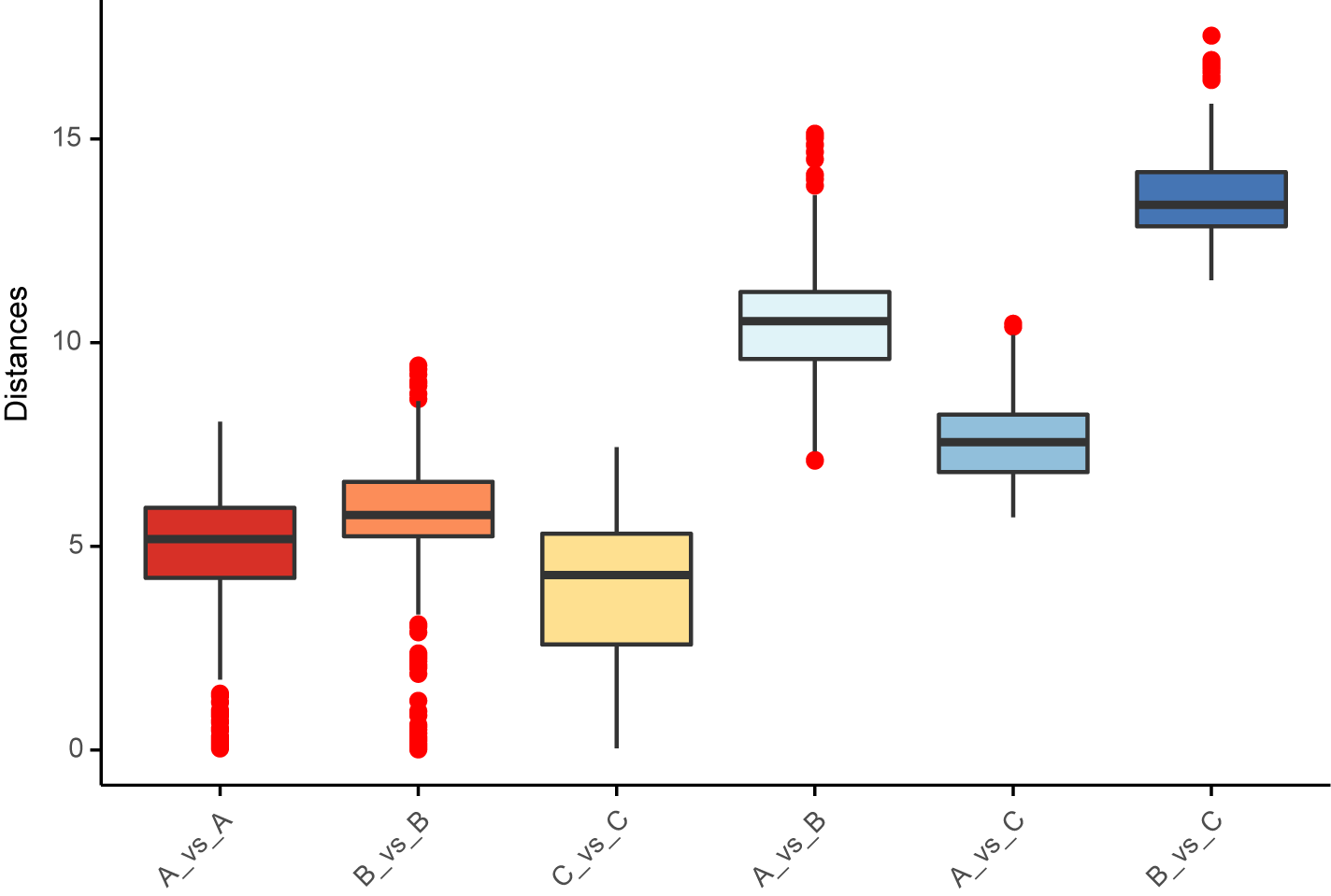


**Fig. S2. Genetic distance distribution among HSF subfamilies.**

Boxplot showing the distribution of pairwise phylogenetic distances among HSF proteins within and between subfamilies A, B, and C. The comparisons include intra-subfamily distances (A vs. A, B vs. B, and C vs. C) and inter-subfamily distances (A vs. B, A vs. C, and B vs. C). The results indicate distinct divergence patterns among HSF subfamilies, supporting the phylogenetic classification presented in Fig. 2. Boxes represent the interquartile range, horizontal lines indicate median values, and red dots denote outliers.
